# Root-knot nematode modulates plant CLE3-CLV1 signaling as a long-distance signal for successful infection

**DOI:** 10.1126/sciadv.adf4803

**Published:** 2023-06-02

**Authors:** Satoru Nakagami, Michitaka Notaguchi, Tatsuhiko Kondo, Satoru Okamoto, Takanori Ida, Yoshikatsu Sato, Tetsuya Higashiyama, Allen Yi-Lun Tsai, Takashi Ishida, Shinichiro Sawa

**Affiliations:** ^1^ Graduate School of Science and Technology, Kumamoto University, Kumamoto 860-8555, Japan.; ^2^ Bioscience and Biotechnology Center, Nagoya University, Nagoya 464-8601, Japan.; ^3^ Graduate School of Bioagricultural Sciences, Nagoya University, Nagoya 464-8601, Japan.; ^4^ Graduate School of Science and Technology, Niigata University, Niigata 950-2181, Japan.; ^5^ Japan Science and Technology Agency, Precursory Research for Embryonic Science and Technology, Saitama, 332-0012, Japan.; ^6^ Department of Bioactive Peptides, Frontier Science Research Center, University of Miyazaki, Miyazaki 889-1692, Japan.; ^7^ Institute of Transformative Bio-Molecules (WPI-ITbM), Nagoya University, Nagoya 464-8601, Japan.; ^8^ Department of Biological Sciences, Graduate School of Science, University of Tokyo, Tokyo 113-0033, Japan.; ^9^ International Research Center for Agricultural & Environmental Biology, Kumamoto University, Kumamoto 860-8555, Japan.; ^10^ International Research Organization for Advanced Science and Technology (IROAST), Kumamoto University, Kumamoto 860-8555, Japan.; ^11^ Institute of Industrial Nanomaterial (IINA), Kumamoto University, Kumamoto 860-8555, Japan.

## Abstract

Plants use many long-distance and systemic signals to modulate growth and development, as well as respond to biotic and abiotic stresses. Parasitic nematodes infect host plant roots and cause severe damage to crop plants. However, the molecular mechanisms that regulate parasitic nematode infections are still unknown. Here, we show that plant parasitic root-knot nematodes (RKNs), *Meloidogyne incognita*, modulate the host CLAVATA3 (CLV3)/EMBRYO SURROUNDING REGION (CLE)–CLV1 signaling module to promote the infection progression. Plants deficient in the *CLE* signaling pathway show enhanced RKN resistance, whereas *CLE* overexpression leads to increased susceptibility toward RKN. Grafting analysis shows that *CLV1* expression in the shoot alone is sufficient to positively regulate RKN infection. Together with results from the split-root culture system, infection assays, and CLE3-CLV1 binding assays, we conclude that mobile root-derived CLE signals are perceived by CLV1 in the shoot, which subsequently produce systemic signals to promote gall formation and RKN reproduction.

## Teaser

Plant parasitic nematode utilizes plant systemic CLE3-CLV1 peptide signaling for their successful infection.

## INTRODUCTION

Long-distance and systemic signals are particularly well developed in plants. These signals help plants to respond to various stresses, which allow them to adapt to a wide range of environments. Systemin, the first peptide hormone isolated from plants, plays a critical role during biotic stress responses (*1*). Systemin is synthesized locally in herbivory wound sites but induces systemic immune responses to protect the entire plant from further herbivore attacks. Long-distance and systemic signals have also been documented to modulate plant-microbe interactions. In the legume plant *Lotus japonicus*, rhizobia-induced root nodulation is suppressed by CLAVATA3 (CLV3)/EMBRYO SURROUNDING REGION (CLE) peptides and their cognate receptor, leucine-rich repeat (LRR) receptor–like kinase HYPER-NODULATION ABERRANT ROOT FORMATION 1 (LjHAR1) (*2*, *3*). This negative-feedback mechanism prevents excessive nodulation that inhibits the growth of the host plants.

The *CLE* genes encode one of the largest families of small signaling peptides conserved among terrestrial plants, with the genome of the model plant *Arabidopsis thaliana* containing 32 *CLE* members annotated to date (*4*–*6*). CLE peptides are typically synthesized as pre-propeptides, which subsequently undergo proteolysis to yield mature bioactive peptides around 12 to 13 amino acids in length. These mature CLE peptides are secreted extracellularly and perceived by plasma membrane-bound LRR receptor proteins, which initiate signal cascades to control stem cell maintenance (*7*). It has been shown that a few CLE peptides translocate from roots to shoots and are recognized by shoot-expressed receptors (*3*, *8*). As the CLE-CLV signaling module is evolutionarily ancient and regulates such basal functions in plants, parasites have evolved to directly manipulate it as part of the infection process. For instance, the cyst nematodes (CNs; *Heterodera* and *Globodera* spp.) have been documented to inject CLE peptide mimics into host plant cells, presumably as a mechanism to positively regulate infection (*9*–*11*). However, little is known about whether the endogenous plant peptide signals regulate nematode infections.

Root-knot nematode (*Meloidogyne incognita*, RKN) is another class of plant-parasitic nematodes known to cause severe yield losses of many crop plants worldwide (*12*). During RKN infections, infective juveniles invade plant roots and migrate to the vascular cylinder, where they induce the formation of specialized feeding sites called galls or root-knots. Each gall contains five to seven nematode-induced giant cells (GCs), which serve as the nutrient source for the nematode until maturity. A recent study has reported that a systemic root-shoot signaling loop that integrates electrical, reactive oxygen species, and jasmonates signals enhances the host plant’s resistance against RKNs (*13*). However, little is known about whether plant endogenous small signaling peptides are involved in nematode infection. Here, we demonstrate that RKN exploits the host’s CLE-CLV1 signaling module to positively regulate their infections.

## RESULTS

### Four *CLE* genes are up-regulated in galls and positively regulate gall formation

**Fig. 1.**
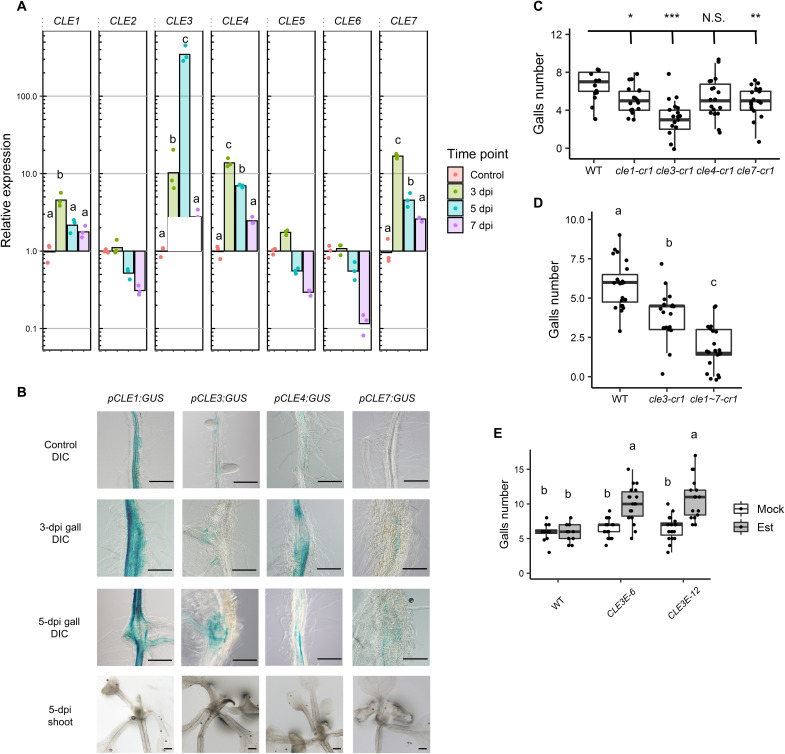
*CLE* genes are up-regulated in galls and positively regulate gall formation. (**A**) qRT-PCR results denoting *CLE1* to *CLE7* expression levels in wild-type (WT) galls at 3, 5, and 7 dpi, values are normalized to expression levels in uninfected roots (*n* = 3 biological replicates). Experiment was repeated three times with similar results. (**B**) Promoter activities of *CLE1*, *CLE3*, *CLE4*, and *CLE7* genes shown as GUS stains at 5 dpi (five individuals were observed with similar results). Top row: Differential interference contrast (DIC) images of uninoculated roots. Second row from the top: DIC images of 3 dpi galls. Second row from the bottom: DIC images of 5 dpi galls. Bottom row: Bright-field images of shoots at 5 dpi. Scale Bars, 200 μm. (**C**) Gall numbers in WT, *cle1-cr1*, *cle3-cr1*, *cle4-cr1*, and *cle7-cr1* at 7 dpi (*n* ≥ 13). Asterisks denote significant differences [*P < 0.05, **P < 0.01, and ***P < 0.001; one-way analysis of variance (ANOVA) followed by Dunnett’s multiple test when compared to WT]. N.S., not significant. (**D**) Gall numbers in WT, *cle3-cr1*, and *cle1~7-cr1* at 7 dpi (*n* ≥ 21). (**E**) Gall numbers in WT, *CLE3E-6*, and *CLE3E-12* transgenic plants treated with 10 μM β-estradiol for 18 hours at 7 dpi (*n* ≥ 12). Est, 10 μM β-estradiol treatment. Alphabets denote significant differences (*P* < 0.05, two-way ANOVA followed by Tukey’s test).

To identify the *CLE* genes involved in RKN infections, transcriptome data from early stage RKN-induced *Arabidopsis* galls were analyzed. Transcripts of 23 *CLE* genes were detected in both uninoculated roots and galls (table S1) (*14*). Of these 23 *CLE* genes, *CLE3* was the most highly up-regulated in galls during all the time points examined (table S1). Since *CLE1*, *CLE2*, *CLE3*, *CLE4*, *CLE5*, *CLE6*, and *CLE7* form a monophyletic group based on their amino acid sequences, these *CLE* genes are predicted to be functionally redundant (*6*, *15*). Recent studies have confirmed that this is the case (*16*, *17*). Quantitative reverse transcription polymerase chain reaction (qRT-PCR) analysis showed that not only *CLE3* but also three of its homologs *CLE1*, *CLE4*, and *CLE7* were also up-regulated in 3 days post-inoculation (dpi) galls (Fig. 1A). The transcript levels of *CLE3*, *CLE4*, and *CLE7* in 5 dpi galls remained higher than in uninoculated roots but did not elevate further in 7 dpi galls (Fig. 1A). In particular, *CLE3* transcript level increased by approximately 100-fold in 5 dpi galls compared to uninoculated roots. Next, we examined the spatial expression patterns of these *CLE* genes. Promoter activities of *CLE1*, *CLE3*, *CLE4*, and *CLE7* were obviously increased in 3 and 5 dpi galls compared to uninoculated roots, whereas none of these promoters were active in the shoots during RKN infection (Fig. 1B). *CLE1*, *CLE3*, *CLE4*, and *CLE7* have been shown to be expressed inside the root cortex (*18*, *19*). Consistent with these findings, promoter activities of *CLE1*, *CLE3*, *CLE4*, and *CLE7* were detected in the inside the cortex, but not the GCs of 5 dpi galls (fig. S1A). These results suggest that *CLE1*, *CLE3*, *CLE4*, and *CLE7* are involved in RKN-induced gall formation.

To evaluate the contribution of individual *CLE* genes in gall formation, we assayed the number of galls in the corresponding loss-of-function *cle* mutants. Gall numbers were reduced significantly in *cle1-cr1*, *cle3-cr1*, and *cle7-cr1* compared to the wild type at 7 dpi but was not changed in *cle4-cr1* (Fig. 1C), suggesting that *CLE1*, *CLE3*, and *CLE7* positively regulate gall formation. In particular, *cle3-cr1* showed the most pronounced gall number reduction among the *cle* mutants tested. To explore the possibility of functional redundancies among the *CLE* genes, we used the *cle1 cle2 cle3 cle4 cle5 cle6 cle7* septuple mutant (denoted as *cle1~7-cr1* hereafter) and evaluated its RKN infection rate. As a result, *cle1~7-cr1* exhibited further reductions in gall numbers compared to *cle3-cr1* (Fig. 1D and fig. S1B). These results suggest that *CLE1* to *CLE7* share overlapping functions and *CLE3* plays a major role in RKN-induced gall formation. Conversely, we also investigated the effects of *CLE3* overexpression on RKN infection efficiency. We generated estradiol-inducible *CLE3* (*p35S:XVE*, *pOlexA:CLE3*) transgenic plants in the wild-type background (denoted as *CLE3E-6* and *CLE3E-12* hereafter; fig. S1C) (*20*). Gall numbers were significantly increased in β-estradiol–treated *CLE3E-6* and *CLE3E-12* plants compared to mock-treated transgenic plants and β-estradiol–treated wild-type plants (Fig. 1E). These results further support the notion that *CLE3* positively regulates gall formation.

### CLV1 is a downstream target of CLE3

**Fig. 2.**
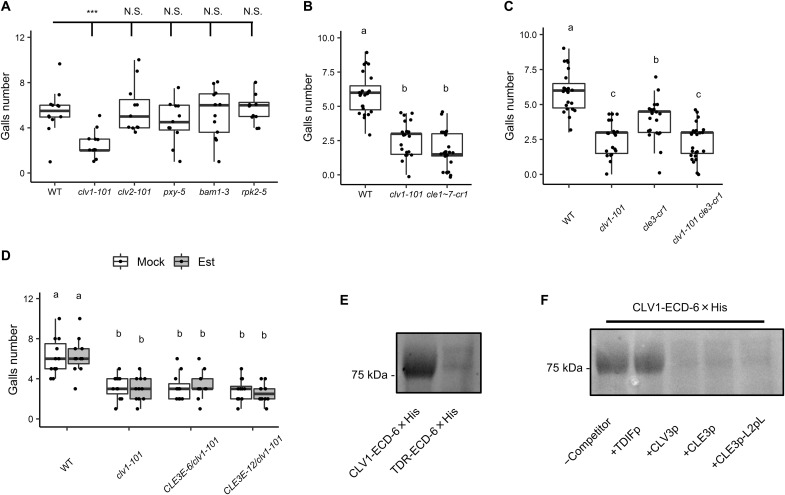
CLE3 peptide functions with CLV1 receptor to facilitate gall formation. (**A**) Gall numbers in WT, *clv1-101*, *clv2-101*, *pxy-5*, *bam1-3*, and *rpk2-5* at 7 dpi (*n* ≥ 11). Asterisk denotes significant difference (***P < 0.001, one-way ANOVA followed by Dunnett’s multiple test when compared to WT). (**B**) Gall numbers in WT, *clv1-101*, and *cle1~7-cr1* at 7 dpi (*n* ≥ 21). (**C**) Gall numbers in WT, *clv1-101*, *cle3-cr1*, and *clv1-101 cle3-cr1* double mutants at 7 dpi (*n* ≥ 21). (**D**) Gall numbers in WT, *clv1-101*, *CLE3E-6/clv1-101*, and *CLE3E-12/clv1-101* plants at 7 dpi that were mock-treated or treated with 10 μM β-estradiol for 18 hours (*n* ≥ 11). (**E**) Photoaffinify labeling assay using CLE3p-L2pL-R10Pra as ligand against CLV1-ECD-6×His or TDR-ECD-6×His. (**F**) Competitive replacement of binding to CLV1-ECD-6×His by TDIFp, CLV3p, CLE3p, and CLE3p-L2pL. Experiments were repeated twice with similar results (E) and (F). Alphabets denote significant differences [*P* < 0.05, one-way ANOVA (B) and )C) or two-way ANOVA (D) followed by Tukey’s test].

Peptide hormones require cognate receptors for signal transduction (*7*, *21*). To identify the receptor(s) that recognize the CLE peptides to regulate gall formation, we performed RKN infection assays using loss-of-function mutants of various putative CLE receptor LRR. *clv1-101* showed reduced gall numbers at 7 dpi, whereas *clv2-101*, *phloem intercalated with xylem* (*pxy-5*), *barely any meristem 1* (*bam1-3*) and *receptor-like protein kinase 2* (*rpk2-5*) did not show significant changes in gall numbers compared to the wild type (Fig. 2A). Taking into account the possible functional redundancy among the LRR receptors, we also examined the gall numbers in double- and triple- LRR mutants by crossing the aforementioned single mutants. Gall numbers decreased in all mutants that carry the *clv1-101*, whereas mutants that are wild type at the *CLV1* gene did not show alterations in gall formation rates compared to the wild type (figs. S1B and S2A). These results indicate that out of all the LRR receptors tested, only *CLV1* is involved in RKN-induced gall formation.

We also examined the roles of CLE1~7 and CLV1 in RKN infection. GC areas were significantly reduced in *clv1-101* (fig. S2, B and C). The numbers of mature females and egg masses from both *cle1~7-cr1* and *clv1-101* plants were also significantly reduced compared to those from the wild type (fig. S2, D and E). These findings suggest that the CLE1~7-CLV1 signaling module specifically regulates GC formation during gall formation, which in turn dictates RKN fecundity.

Next, we studied the functional relationship between CLV1 and the CLEs in RKN infection. *clv1-101* shows reduced gall numbers similar to *cle1~7-cr1*, suggesting that *CLV1* may be functionally synonymous with the *CLEs*, and that *CLV1* may be required to transduce the *CLE1~7* signals during gall formation (Fig. 2B). To investigate the genetic interactions between *CLV1* and *CLE*, we isolated the *clv1-101 cle3-cr1* double mutant and the *CLE3* estradiol–inducible transgenic plants in the *clv1-101* background (*CLE3E-6/clv1-101* and *CLE3E-12/clv1-101*; fig. S1D). Gall numbers in *clv1-101 cle3-cr1* were not significantly different from *clv1-101* (Fig. 2C). Meanwhile, gall numbers in β-estradiol–treated *CLE3E-6/clv1-101* and *CLE3E-12/clv1-101* plants were comparable to that of *clv1-101* (Fig. 2D). Since neither knocking out nor overexpressing *CLE3* alters the *clv1-101* phenotype, these results imply that *CLE3* and *CLV1* function in the same pathway, and the *clv1-101* mutation is epistatic over *CLE3* overexpression in terms of RKN infection facilitation.

Next, we asked whether CLV1 is the cognate receptor that binds CLE3. We conducted CLE3-CLV1 binding assay using a combination of photoaffinity labeling and Cu-catalyzed azide alkyne cycloaddition (CuAAC) reaction. To examine the biological activities of synthetic CLE3 peptides, we applied CLE3 synthetic peptides to *clv3-8* seedlings to determine whether they can rescue the enlarged shoot apical meristem (SAM) defect in *clv3-8* (*22*). We first determined the biologically active form of the CLE3 peptide, since the mature CLE peptides have been hypothesized to be either in the 12– or 13–amino acid form (*7*). The 12–amino acid form of CLE3 was able to reduce the *clv3-8* SAM size, whereas the 13–amino acid form did not, suggesting that the mature CLE3 peptide is likely in the 12–amino acid form (fig. S3, A to C). Then, we synthesized variants of the 12–amino acid CLE3 peptide with amino acid residues systematically replaced with propargylglycine (Pra) to determine the optimal alkyne cycloaddition site (fig. S3A). As a result, only CLE3p-R10Pra, where Arg^10^ was substituted with Pra, retained biological activities at a concentration of 1 μM in controlling SAM size (fig. S3, B and C). Therefore, CLE3-L2pL-R10Pra, where Leu^2^ was substituted with photoleucine to cross-link with the CLV1 receptor for photoaffinity labeling, was selected as the ligand for the binding assay. The extracellular domains of CLV1 and TRACHEARY ELEMENT DIFFERENTIATION FACTOR (TDIF) RECEPTOR (TDR; also known as PXY) were fused with 6×His tags at the C termini, and then expressed in Sf9 insect cells (CLV1-ECD-6×His and TDR-ECD-6×His). The variants can be detected as approximately 80-kDa proteins from the media (fig. S3D). Purified CLV1-ECD-6×His and TDR-ECD-6×His were photoaffinity labeled with CLE3-L2pL-R10Pra, and then labeled with BODIPY (BDP) azide, which was used as a fluorescent probe, via the CuAAC reaction. After SDS–polyacrylamide gel electrophoresis and detection of BDP fluorescence, the CLV1 protein was detected as an 80-kDa band, while TDR was hardly detectable (Fig. 2E and fig. S3E). The BDP signal was attenuated with the addition of excess CLV3p, CLE3p, and CLE3p-L2pL, but not with TDIFp (Fig. 2F and fig. S3E). These results indicate that CLE3 directly binds to CLV1. Together, CLV1 is a cognate receptor of CLE3 in RKN infection regulation.

### *CLV1* expression was down-regulated in roots but unchanged in shoots during infection

**Fig. 3.**
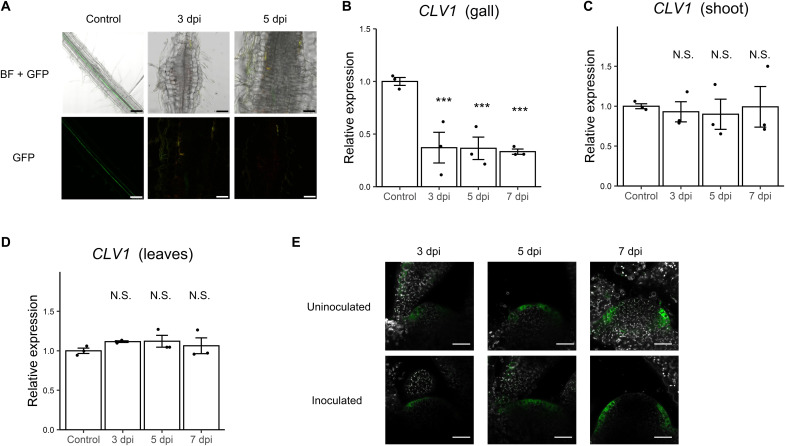
Expression pattern of *CLV1*. (**A**) CLV1-GFP localization in uninoculated primary roots and galls at 3 and 5 dpi (six individuals were observed with similar results). (**B** to **D**) CLV1 expression level at 3, 5, and 7 dpi in galls (B), shoots (C), and leaves (D) determined with qRT-PCR (*n* = 3 biological replicates). Tissues from uninfected plants were used as controls. Experiment was repeated three times (B) and (C) or twice (D) with similar results. (**E**) Lateral optical sections of *CLV1-GFP* vegetative SAM with or without RKN inoculation after 3, 5, and 7 days (five individuals were observed with similar results). Scale bars, 100 μm (A) and 20 μm (E). Asterisks denote significant differences (***P < 0.001, one-way ANOVA followed by Dunnett’s multiple test when compared to WT).

*CLV1* and its known ligands are known to be expressed in regions adjacent from one another, consistent with their roles as cognate receptor and ligands in short-distance signaling (*23*–*26*). Furthermore, CNs have been shown to inject CLE peptide–mimic effectors into plant cells to stimulate CLV1 signaling locally, while the up-regulation of *CLV1* in the syncytia has been shown to be important for CN infection (*9*–*11*). Therefore, we investigated whether *CLV1* expression is similarly enhanced in RKN-induced galls. *pCLV1:CLV1-green fluorescent protein* (*CLV1-GFP*) transgenic plant was used to monitor CLV1 protein level during gall formation. Unexpectedly, CLV1-GFP signals were hardly detected in developing galls (Fig. 3A). Consistent with this finding, *CLV1* transcript levels in galls were also reduced compared to uninfected roots (Fig. 3B). In contrast, *CLV1* expression levels in the shoot, leaves, and SAM remain unchanged regardless of RKN infection (Fig. 3, C to E). These lines of evidence suggest that, unlike CN, RKN infections do not induce *CLV1* expression in the feeding site while bringing up the question of where the CLE peptides induced by RKN infection are perceived by CLV1, if not in the galls.

### CLE3 and CLV1 function in long-distance signaling during RKN-induced gall formation

**Fig. 4.**
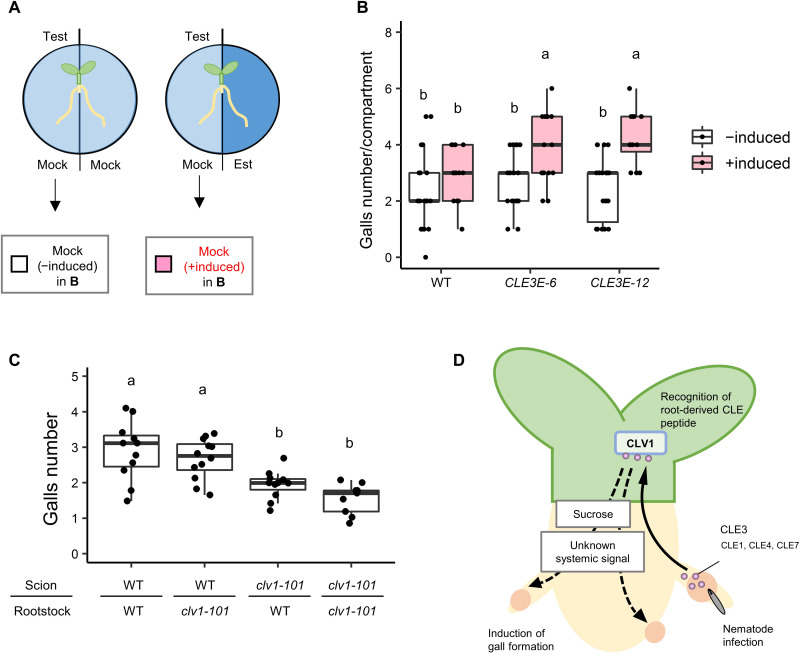
CLE3 signals over long distances to mediate systemic effects. (**A**) Experimental design of infection assay the split-root culture system for partial *CLE3* expression induction. For the +induced condition, one compartment of split-root system contains medium supplemented with 10 μM β-estradiol to induce *CLE3* expression, whereas the other compartment contains mock-treated medium without β-estradiol. For the uninduced control condition, both sides of the system contain mock-treated medium without β-estradiol. Only galls from the Mock side of the system were counted for analyses. (**B**) Gall numbers per compartment in WT, *CLE3E-6*, and *CLE3E-12* transgenic plants at 7 dpi in the split-root culture system (*n* ≥ 15; see also figs. S4 and S5 and table S2). Only gall numbers from the left compartment of the petri dish as shown in (A) were shown in (B). Alphabets denote significant differences (*P* < 0.05, two-way ANOVA followed by Tukey’s test). (**C**) Gall numbers in the grafted chimeric plants at 7 dpi (*n* ≥ 9). Alphabets denote significant differences (*P* < 0.05, one-way ANOVA followed by Tukey’s test). (**D**) Model of CLE-CLV1 signaling module functioning early in the RKN infection process. After RKN infection, *CLE3* and its homologous genes are up-regulated in galls, which in turn the CLE peptides transfer from galls to shoot, where CLV1 recognizes the CLE peptides. Unknown systemic signal(s) (possibly sucrose) induces gall formation downstream of CLV1.

CLE peptides have been documented to be involved in organ-to-organ and cell-to-cell communication events (*2*, *8*). Consistent with this function, CLE peptides have been detected from xylem, suggesting that they are capable of traveling long distances (*27*, *28*). In addition, our expression analysis showed that RKN-induced the up-regulation of *CLE3* genes occur in galls, but not in shoots (Fig. 1B). These lines of evidence highlight the possibility that CLE3 and its homologous peptides may be synthesized in the roots and perceived in the shoots to regulate gall formation. To evaluate this hypothesis, we used a split-root culture system to conduct a modified RKN infection assay, where the roots from a single plant are divided onto two compartments of a petri dish and can be treated independently (fig. S4A) (*29*). Induction of *CLE3* transcripts and H2B-sfGFP by β-estradiol was confirmed to occur only in the induced compartment of the roots (fig. S4, B and C). We then verified that β-estradiol treatment enhances gall formation in *CLE3E-6* and *CLE3E-12*, while mock-treated *CLE3E-6* and *CLE3E-12* and β-estradiol–treated wild-type plants did not show altered gall formation rates when both compartments receive the same treatment (fig. S5, B to D, and table S2). When only one compartment of *CLE3E-6* or *CLE3E-12* seedlings was treated with β-estradiol, gall numbers increased in both compartments, including the compartment where *CLE3* was not induced (Fig. 4, A and B; fig. S5, A and D; and table S2). These results confirm that *CLE3* acts systemically to promote gall formation.

In legumes, root-derived CLE peptides and their cognate receptors regulate rhizobia nodulation through long-distance signaling (*2*, *3*, *30*–*32*). Therefore, we hypothesize that the CLE-CLV1 module may similarly control gall formation via long-distance signaling. To test this hypothesis, we established chimeric plants by reciprocally grafting the scions and the rootstocks of wild-type and *clv1-101* plants, and then subjected these chimeric plants to RKN infection. Chimeric plants with wild-type scion and *clv1-101* rootstock did not show significant changes in gall numbers. On the other hand, chimeric plants with the reverse setup of *clv1-101* scion and wild-type rootstock exhibited a significant decrease in gall numbers compared to the mock-grafted wild-type plant, but similar to the mock-grafted *clv1-101* plants (Fig. 4C). This confirms that CLV1 expression in the shoot alone is sufficient to facilitate gall formation, while CLV1 expression in the roots is not (Fig. 4D). These results support our hypothesis that the CLE peptides regulate gall formation by functioning as long-distance signals recognized by shoot-expressed CLV1 (Fig. 4D). Recent studies have shown that *CLE2* and *CLE3* are up-regulated during sucrose deficiency, and *CLE1~7* negatively affects root sucrose levels (*28*, *33*). In addition, CLE2 peptides have been shown to be translocated from root to shoot to regulate *SUCROSE-PROTON SYMPORTER 2* (*SUC2*) expression (*28*). These findings suggest that RKN may regulate root sucrose levels through CLE-CLV1 signaling to aid its own infection. We therefore tested whether exogenous sucrose can rescue the *clv1-101* and *cle1~7-cr1* gall formation defects. Gall numbers were significantly increased in plants of all genotypes with 3 and 15 mM sucrose supplement compared to untreated plants (fig. S6). Moreover, 15 mM sucrose supplement restored gall formation defects of *clv1-101* and *cle1~7-cr1* back to the wild-type level (fig. S6). This result suggests that CLE-CLV1 signaling likely regulates gall formation by promoting root sucrose status through systemic signals (Fig. 4D).

## DISCUSSION

Recently, CLEs and other CLE-like peptides have been recognized as an important class of signaling molecules in plant-parasitic nematode pathologies. CNs are known to secrete CLE peptide mimics as effectors, which are perceived in the root by CLV receptor homologs, and are pivotal for CN infections (*34*). A recent study has reported that CN CLE peptides injected into plant cell cytoplasm can be translocated from the cell to the apoplast, and this process depends on the variable domain of CN CLE effector proteins (*35*). On the other hand, CLE-like motifs have been identified in RKN proteins, suggesting that RKN may similarly deploy CLE peptide mimics for host manipulation (*36*, *37*). However currently, whether these RKN CLE-like proteins actually function as CLE-mimics at all during infection remains unclear. For example, *Megalaima incognita* 16D10 is one such putative effector protein that contain CLE-like motif, and silencing of *Mi16D10* reduces RKN infectivity (*36*, *38*). However, Mi16D10 was only shown to bind nuclear SCARECROW-LIKE (SCL) proteins, and, currently, no evidence suggests that Mi16D10 can bind LRR receptors, thus reducing the likelihood of Mi16D10 being a bona fide CLE peptide, at least in a traditional sense. In addition, currently, all known RKN CLE–like proteins lack the variable domain required to translocate mature CLE peptides to the apoplast, which is present in CN CLE–mimic effectors. So far, strong candidates for RKN CLE–mimic effectors remain lacking. Several studies have reported marked differences between transcriptional profiles of CN and RKN feeding sites (*39*–*43*). These findings suggest that RKN and CN may use different strategy to establish feeding sites. Therefore, we hypothesized that RKN may use the host plant’s endogenous CLE peptides instead. This study highlights the importance of endogenous plant CLE signaling during RKN infection. Our findings suggest that the CLE-CLV1 signaling module regulates RKN infection through a two-step signaling mechanism (Fig. 4D). In the first step, several *CLE* genes are locally up-regulated in roots upon RKN infection. These peptide ligands then ascend to the shoot and are probably perceived by CLV1 there to establish long-distance signaling. Since knocking out neither *CLE1~7* nor the CLV1 showed abnormalities in root growth and RKN invasion frequency, the CLE-CLV1 module likely functions after the RKN invasion (fig. S7). In the second step, CLV1 signal perception leads to the production of an unknown putative shoot-derived signal that positively modulates gall formation systemically. Considering that exogenous sucrose treatment rescues the gall formation defects in *cle1~7-cr1* and *clv1-101* mutants, it is possible that a shoot-derived signal increases the root sucrose level. We also cannot eliminate the possibility that the signal is sucrose itself. This signaling network may benefit RKN by encouraging repeated infections on the same host plant, as the systemic CLE-CLV1 signaling induced by the first RKN to infect the plant early can benefit gall formation for other RKN that infect the same host later in time. To further investigate this mechanism, next, we plan to investigate how these endogenous CLEs are induced during RKN infection, whether CN CLE mimics also translocate over long distances, and what are the downstream targets of the CLE-CLV1 signaling module during RKN infection.

The CLE-CLV signaling module has been shown to regulate lateral root development and shoot regeneration, and, in these cases, the signal is assumed to be local (*16*, *17*, *23*). On the other hand, our findings showed that CLE-CLV1 signaling can span long distances and act systematically. Therefore, the functions of CLE peptides may be different depending on their destination tissues, although they are perceived by the same receptors. LjHAR1 has been shown to regulate both nodulation and lateral root formation, although only LjHAR1 expressed in the shoots is involved in nodule formation (*44*–*46*). In addition, *Medicago truncatula* LRR receptor–like kinase COMPACT ROOT ARCHITECTURE 2 (MtCRA2) has been shown to locally control lateral root formation but systemically control symbiotic nodule formation (*47*). These lines of evidence are consistent with our results that CLV1 can function in long-distance signaling for specific functions, namely, gall formation regulation. On the other hand, a recent study has reported that LjHAR1 controls root development locally and systemically (*44*). In addition, *Arabidopsis* C-TERMINALLY ENCODED PEPTIDE RECEPTOR 1 (CEPR1) inhibits lateral root growth by both short- and long-distance signaling (*48*, *49*). Considering these findings, RKN may use CLE-CLV1 signaling, which inhibits root growth, as a means to redirect resources to gall development. If this is the case, then gall formation efficiency may be improved by systematically suppressing lateral root development through CLE-CLV1 signaling. To eliminate this confounding factor, 7-day-old seedlings with only primary roots were used in this study to exclude the influences from lateral root development. We did not observe root development alterations caused by *CLE3* and *CLV1* manipulation in the split-root and grafting experiments (fig. S8); thus, we could not conclude whether CLE-CLV1 signaling to regulate lateral root development under our experimental conditions. Nevertheless, it would be interesting to examine whether the CLE-CLV1 signaling module regulates lateral root development through local and/or systemic signaling.

It has been revealed that the transcript level of the *LjHAR1* is not affected by rhizobial colonization (*50*). Similarly, *CLV1* transcript level did not show a significant change in the shoot, leaves, and SAM upon RKN infection (Fig. 3). These receptors of long-distance signals in response to biotic stresses may be stable proteins that do not require further strengthening of the signals, and they may not use feedback mechanisms to regulate their own expression. On the other hand, *CLV1* expression was down-regulated in galls upon RKN infections (Fig. 3, A and B). Araya *et al*. (*23*) have proposed that transcript levels of *CLE1*, *CLE3*, *CLE4*, and *CLE7* may be suppressed by CLV1. Hence, the up-regulation of *CLE* genes in galls observed during RKN infection may in part be explained by the lifting of CLV1 suppression. It appears that the same CLE signaling pathway may even be regulated with different mechanisms in different tissues and signaling contexts.

CLE signaling has been shown to regulate lateral organ formation during nitrogen deficiencies (*23*, *51*–*53*). In legumes, *CLE* induction after rhizobial infection is directly regulated by NODULE INCEPTION (NIN)-LIKE PROTEIN (NLP) transcription factors, which are known as key regulators in the nitrate-induced pleiotropic control of root nodule symbiosis (*51*–*53*). In addition, a recent study has reported that the CLE-CLV1 signaling module also regulates lateral root development during sulfur deficiency (*54*). We therefore checked whether the expression levels of nitrate-responsive genes have changed in RKN-induced galls to evaluate the relationship between RKN infection and nitrate response (*14*). As a result, the expression levels of several nitrate-responsive genes were altered in the galls compared to uninfected roots (fig. S9). In addition, sucrose represses *CLE2* and *CLE3* expression, while *CLE*s expressed in roots promote *SUC2* expression in shoots, which in turn increases sucrose levels in roots (*28*, *33*). We also showed that sucrose rescues CLE-CLV1 signaling defects during gall formation (fig. S6). Considering that the *suc2* mutation delays RKN development (*55*), *CLE1*, *CLE3*, *CLE4*, and *CLE7* up-regulation in galls may facilitate infection by increasing root sucrose levels. Thus, it appears that both pathogen infections and commensal colonization in the roots are associated with the host’s nutrient level and abiotic stress.

Here, we demonstrate that pathogens manipulate the host’s endogenous peptide signal in land plants as part of the infection process. Furthermore, our results also shed light on how peptides may function as long-distance signaling molecules in interspecific interactions. Wang *et al*. (*13*) have reported a systemic root-shoot-root signaling loop that enhances resistance against RKN by integrating electrical, reactive oxygen species, and jasmonate signals. A recent study has also shown that leaf herbivory suppresses RKN infection via the jasmonate signaling pathway in shoot-to-root signaling (*56*). Therefore, plant systemic signals can potentially control both the susceptibility and resistance against RKN infection. RKNs are known to be nonselective with their hosts and can infect many plant species. The manipulation of conserved plant endogenous signaling pathways may thus be a key factor that contributes to RKN’s wide host range, and the ability to flourish in multiple complex ecosystems over the world.

## MATERIALS AND METHODS

### Plant materials and growth conditions

All *A. thaliana* plants except for *CLV1-GFP* used in this study are of the Columbia ecotype background. The *CLV1-GFP* is of the *clv1-4* background (*23*). The mutant lines *clv1-101* (*CS858348*), *clv2-101* (*GK686A09*), *bam1-3* (*SALK_015302*), *bam3-2* (*SALK_044433*), *pxy-5* (*SALK_002910*), *rpk2-5*, *cle1-cr1*, *cle3-cr1*, *cle4-cr1*, *cle7-cr1*, and *cle1~7-cr1* have been previously described (*11*, *28*, *57*–*60*). The *clv1-101 cle3-cr1* double mutant was generated by crossing *clv1-101* and *cle3-cr1*. The *pxy-5 clv1-101 clv2-101* triple mutant was generated by crossing *pxy-5 clv1-101* and *clv2-101*. The *CLE3E-6/clv1-101* and *CLE3E-12/clv1-101* plants were generated by crossing *clv1-101* and *CLE3E-6* or *CLE3E-12*. The reporter lines *CLV1-GFP*, *pCLE1::GUS*, *pCLE3::GUS*, *pCLE4::GUS*, and *pCLE7::GUS* have been previously described (*19*, *23*).

For the construction of pER8-GW-H2B-sfGFP, the *Arabidopsis* H2B gene sequence (*61*) was amplified by PCR using the primer set H2B_entry_F and H2B_entry_R, and the sfGFP coding sequence was PCR-amplified from the sfGFP-N1 (*62*) (Addgene, #85042) using the primer set mScarlet_F and sfGFP-R. These two PCR products were cloned in pDONR221 using In-Fusion HD Cloning Kit (TaKaRa) to generate the H2B-sfGFP construct. The H2B-sfGFP was recombined into pER8-GW (*63*) using the Gateway LR Clonase II Enzyme Mix (Thermo Fisher Scientific). The resultant pER8-GW-H2B-sfGFP binary vector was used to transform Col-0 plants using the floral dipping method. The primer sequences are listed in table S3.

For the construction of *CLE3* β-estradiol induced lines, *CLE3* ORF was cloned into the XhoI and SpeI sites of pER8 using the In-Fusion kit (Clontech). Primers used for the constructs are listed in table S3.

*Arabidopsis* seeds were stratified for 2 days at 4°C in the dark, and then allowed to germinate and grown for 5 days on 0.25 × Murashige and Skoog (MS) salt mixture (Sigma-Aldrich), 0.5% (w/v) sucrose, and 0.6% (w/v) gellan gum at pH 6.4 under continuous light (70 μmol s^−1^ m^−2^) at 23°C. For infection assay, seedlings were grown for 7 days on 0.5 × MS and 0.6% (w/v) gellan gum without sucrose at pH 5.7 under continuous light at 23°C. Sucrose reduces grafting success rates and was therefore omitted in media used in grafting experiments described below. To ensure uniformity for all growth conditions in this study, except for fig. S6, we omitted sucrose from the media used in all of the experiments.

### Nematode preparation and inoculation

*M. incognita* J2 larvae were prepared aseptically as described previously (*64*). Five- or 7-day-old *Arabidopsis* seedlings were inoculated with approximately 80 nematodes per plant and incubated under the short-day condition (8-hour light/16-hour dark) (50 μmol s^−1^ m^−2^) at 25°C. The roots of seedlings were covered with black paper to simulate below-ground conditions. Galls formed on a primary root were counted at 7 dpi except for split-root and grafting experiments. For infection assay shown in fig. S6, *Arabidopsis* seedlings were grown for 7 days on 0.5 × MS, 0.6% (w/v) gellan gum without sucrose, pH 5.7 under continuous light (70 μmol s^−1^ m^−2^) in 23°C. Seedlings were then transferred to MS media containing 0, 3, or 15 mM sucrose and inoculated with approximately 80 RKNs per plant. RKN-inoculated plants were grown for 7 days under the short-day condition (8-hour light/16-hour dark) at 25°C. Mannitol was used as an osmotic control in 0 and 3 mM sucrose treatments.

### Fluorescent microscopy

CLV1-GFP fluorescence in roots and galls was analyzed using a TCS SPE microscope (Leica) using the 488-nm laser for excitation and a 505-550–nm band-pass filter for GFP detection.

A confocal laser scanning microscope (LSM780-DUO-NLO, Zeiss) was used to observe CLV1-GFP in the SAM. Seedlings had their leaves and leaf primordia carefully removed, and then mounted in water. A 40× water-immersion objective lens (LD C-Apochromat, numerical aperture 1.10) was used. Z-sections (1.2-μm interval, 14 planes) were imaged with a 488-nm laser and acquired an emission spectrum of 504 to 653 nm using GaAsP detectors with an 8.8-nm bandwidth for a lambda stack, which were used for linear unmixing to separate autofluorescence. Optical maximum-intensity projections were generated from all 14 z-stack images (Fig. 3E). GFP signal in the root was used as a reference spectrum for spectral unmixing. The images were analyzed using the ZEN 2010 software (Zeiss).

### Gene expression analysis

Total RNA was extracted using the RNeasy Plant Mini Kit (Qiagen), and then treated with Recombinant DNase I (Takara) according to the manufacturer’s instructions. First-strand cDNA was synthesized from 300 ng of total RNA using the PrimeScript RT Master Mix (Takara). Transcript levels of target genes were assayed using the FastStart Essential DNA Green Master (Roche) and LightCycler 480 system (Roche). The thermal cycler program was 95°C for 5 min followed by 55 cycles of 95°C for 10 s, 60°C for 10 s, and 72°C for 10 s. *GLYCERALDEHYDE-3-PHOSPHATE DEHYDROGENASE OF PLASTID 1* (*GAPDH*) (Figs. 1A and 3B and figs. S1, C and D, and S4B) or β*-TUBLIN 4* (*TUB4*) (Fig. 3, C and D) was used as the internal control, and relative expression levels were calculated using the 2-ΔΔCT method. The sequences of primers used are listed in table S3. Each of the three biological replicates was performed in technical duplicate or triplicate.

### Split-root culture system

*Arabidopsis* seedlings were grown for 7 days on 0.5 × MS, 0.6% (w/v) gellan gum, pH 5.7 under continuous light (70 μmol s^−1^ m^−2^) at 23°C. To induce lateral root development, two-thirds of the primary roots were cut off, and the seedlings were grown for an additional 6 days. Seedlings displaying healthy root growths were transferred onto 9-cm two-compartment petri dishes (AS ONE) containing medium supplemented with 0.01% dimethyl sulfoxide (DMSO) (Mock) in one compartment and medium supplemented with 10 μM β-estradiol in 0.01% DMSO (Est) in the other, and grown for an additional 18 hours. Mock/Mock and Est/Est plates were used as positive and negative controls, respectively. Seedlings were then transferred onto a two-compartment Mock/Mock plate to terminate estradiol induction and inoculated with approximately 80 RKNs per compartment. RKN-inoculated plants were grown for 7 days under the short-day condition (8-hour light/16-hour dark) at 25°C.

### *Arabidopsis* seedling grafting

*Arabidopsis* seeds were stratified for 3 days at 4°C in the dark, and then allowed to germinate and grown for 5 days on 0.5 × MS, 0.6% (w/v) gellan gum, pH 5.7 under continuous light (100 μmol s^−1^ m^−2^) at 22°C. Hypocotyl wedge grafting was performed essentially as described (*65*). Grafted plants were cultured for 6 days under continuous light (50 μmol s^−1^ m^−2^) at 27°C. Then, grafted plants were grown for 4 days under continuous light at 22°C and inoculated with approximately 80 RKNs per plant. RKNs-inoculated plants were grown for 7 days under the short-day condition (8-hour light/16-hour dark) at 25°C.

### RNA-seq analysis

RNA-seq data of *Arabidopsis CLE* expression in RKN-induced galls were obtained from (*14*). Reads were aligned with the *Arabidopsis* genome (Tair10) and reads per gene were counted using QuasR (*66*). Differentially expressed *CLE* genes (false discovery rate < 0.05) were identified by TCC (*67*). Data were normalized to transcript levels from uninoculated roots.

### Microscopy

For histological GUS staining, RKN-induced galls, infected whole seedlings, and uninfected roots at 3, 5, and 7 dpi were fixed in 90% (v/v) acetone for 1 hour at 25°C, and then transferred into GUS solution [100 mM NaPO_4_ buffer (pH 7.0), 10 mM EDTA (pH 8.0), 3 mM potassium ferricyanide, 3 mM potassium ferrocyanide, 0.01% Triton X-100, and 5-bromo-4-chloro-3-indolyl-β-d-glucuronidase (0.5 mg/ml)]. Samples were incubated at 37°C overnight. The reaction was stopped with Carnoy’s solution [90% (v/v) ethanol and 10% (v/v) acetic acid], and samples were mounted in chloral hydrate solution (8 g of chloral hydrate, 2 ml of ultrapure water, and 1 ml of glycerol). Samples were imaged with an Axio Imager M1 microscope (Carl Zeiss) mounted with a DP71 digital camera (Olympus). GUS-stained samples were also used for histological analysis by embedding in 4% low-melting agarose, and then sectioned at 50-μm thickness with a VT1000S vibratome (Leica).

For gall cross-section histological analysis, galls were dissected and transferred into 2% glutaraldehyde in 20 mM cacodylate buffer, pH 7.4, and vacuum-infiltrated for 10 min twice, and then incubated at 4°C overnight. Samples were dehydrated in a graded ethanol series and embedded in Technovit 7100 (Kulzer) according to the manufacturer’s protocol. Sample blocks were sectioned to 5-μm thickness using an ultramicrotome (LEICA RM2255, Leica) and stained with 0.01% (w/v) toluidine blue O (WALDECK) containing 1% (w/v) sodium borate decahydrate (Nacalai) for 2 min. All samples were rinsed in deionized water for 1 min. After drying, sections were mounted in EUKITT (O. Kindler). Samples were imaged with an Axio Imager M1 microscope (Carl Zeiss) mounted with a DP71 digital camera (Olympus). Lengths and areas were quantified using ImageJ. For analysis of primary root length, vertically grown 7-day-old seedlings were scanned (GT-X830, EPSON); then, the images of roots were traced by ImageJ.

For RKN acid fuchsin staining, roots of RKN-infected seedlings at 2 dpi were washed in deionized water and transferred in 1% (v/v) sodium hypochlorite for 10 to 30 min. Tissues were washed with deionized water twice, and then boiled in 30-fold diluted acid fuchsin stock solution [250 ml of acetic acid, 750 ml of water, 0.035% (w/v) acid fuchsin] for 10 min. Cooled samples were washed with deionized water twice, and then incubated in acidified glycerol (100 μl of 6 M hydrochloric acid and 500 ml of glycerol) for 2 to 15 min at 95°C. Samples were then mounted in acidified glycerol and imaged with an Axio Imager M1 microscope (Carl Zeiss) and a DP71 digital camera (Olympus). Stained RKNs inside the tissues were counted.

### Preparation of synthetic peptides

The CLV3, TDIF, and CLE3 peptides were prepared at 1-mg scale (DGpeptides). Pra- and photoleucine-substituted variants of the CLE3-12aa form were synthesized by solid-phase peptide synthesis with a peptide synthesizer (Initiator^+^ SP Wave, Biotage). Deprotection and purification of the peptides were carried out as previously described (*68*).

### CLE peptide SAM-reduction bioassay

Wild-type and *cle3-8* seeds were sown on B5 liquid medium supplemented with 1% sucrose and synthetic CLE3 peptides in 12-well plates (2 ml of medium and 10 to 20 seeds per well) and stratified for 2 days. Seeds were then allowed to germinate under continuous light (70 μmol s^−1^ m^−2^) at 23°C for 7 days. Seedlings were mounted in chloral hydrate solution. SAM was defined as the area above a straight line drawn between the basal edges of the two opposing leaf primordia in a micrograph cross section. SAM areas were quantified using ImageJ.

### Expression and purification of CLV1 and TDR recombinant proteins

Coding regions of the CLV1 and TDR extracellular domains (residues 1 to 628 and 1 to 631, respectively) translationally fused with C-terminal 6×His tags were amplified by PCR from *Arabidopsis* cDNA extracted from 7-day-old wild-type seedlings. DNA fragments were cloned in pPSC8 to generate pPSC8-CLV1 and pPSC8-TDR. Baculovirus-mediated transfection of Sf9 insect cells with pPSC8-CLV1 and pPSC8-TDR, validation of protein expression, and preparation of culture cells was performed by Fujifilm Wako Pure Chemical Industries Ltd. The medium was collected and adjusted to pH 8.0, and then centrifuged at 10,000*g* for 10 min at 4°C. The supernatant was filtered by a 0.22-μm polyvinylidene difluoride membrane filter at 4°C. The supernatant was incubated with cOmplete His-Tag Purification Resin (Sigma-Aldrich) at 4°C for 1 hour, and the resin was washed twice with wash buffer [50 mM sodium phosphate (pH 8.0), 500 mM NaCl, and 10 mM imidazole]. The protein was then eluted with elution buffer [50 mM sodium phosphate (pH 8.0), 500 mM NaCl, and 400 mM imidazole]. Primers used for the constructs are listed in table S3.

### On-column photoaffinity labeling and CuAAC reaction

Affinity-purified protein was photoaffinity-labeled as previously described (*69*). The labeled proteins were eluted with the above-described elution buffer and collected using centrifugal filter units (3 K MWCO; Millipore). The buffers of the extracts were exchanged by ultrafiltration to 50 mM sodium phosphate (pH 8.0) at 4°C to remove the imidazole. 2× CuAAC reaction buffer [50 mM sodium phosphate (pH 8.0), 100 μM CuSO_4_, 500 μM tris(3-hydroxypropyltriazolylmethyl)amine (THPTA), 2 mM aminoguanidine, 50 μM BDP FL-PEG5-azide (BroadPharm), and 5 mM sodium ascorbate] was added to the extracts. After incubation at 4°C for 10 min, the solutions were exchanged by ultrafiltration to 50 mM sodium phosphate (pH 8.0) at 4°C. The samples were loaded onto SuperSep Ace, 7.5% gel (Wako), and separated according to the manufacturer’s protocol. The gels were analyzed using a gel analyzer (Molecular Imager FX Pro, BIO-RAD).

### Statistical information

Significant differences were determined by Dunnett’s multiple test, Tukey-Kramer test, or two-sided Student’s *t* tests, as specified in the respective figure legends along with significance cutoff, sample sizes, and the numbers of replicates. Central lines indicate the median and variation indicates the interquartile range in box plots.

## Supplementary Materials

### This PDF file includes:

Figs. S1 to S9 Tables S1 to S3 References

## Notes

View/request a protocol for this paper from *Bio-protocol*.

## References

[1] G. Pearce, D. Strydom, S. Johnson, C. A. Ryan, A polypeptide from tomato leaves induces wound-inducible proteinase inhibitor proteins. Science 253, 895–897 (1991). 17751827 10.1126/science.253.5022.895

[2] S. Okamoto, E. Ohnishi, S. Sato, H. Takahashi, M. Nakazono, S. Tabata, M. Kawaguchi, Nod factor/nitrate-induced *CLE* genes that drive HAR1-mediated systemic regulation of nodulation. Plant Cell Physiol. 50, 67–77 (2009). 19074184 10.1093/pcp/pcn194

[3] S. Okamoto, H. Shinohara, T. Mori, Y. Matsubayashi, M. Kawaguchi, Root-derived CLE glycopeptides control nodulation by direct binding to HAR1 receptor kinase. Nat. Commun. 4, 2191 (2013). 23934307 10.1038/ncomms3191

[4] J. M. Cock, S. McCormick, A large family of genes that share homology with *CLAVATA3*. Plant Physiol. 126, 939–942 (2001). 11457943 10.1104/pp.126.3.939 PMC1540125

[5] K. Oelkers, N. Goffard, G. F. Weiller, P. M. Gresshoff, U. Mathesius, T. Frickey, Bioinformatic analysis of the CLE signaling peptide family. BMC Plant Biol. 8, 1 (2008). 18171480 10.1186/1471-2229-8-1 PMC2254619

[6] Y. Ito, I. Nakanomyo, H. Motose, K. Iwamoto, S. Sawa, N. Dohmae, H. Fukuda, Dodeca-CLE peptides as suppressors of plant stem cell differentiation. Science 313, 842–845 (2006). 16902140 10.1126/science.1128436

[7] L. Y. Yamaguchi, T. Ishida, S. Sawa, CLE peptides and their signaling pathways in plant development. J. Exp. Bot. 67, 4813–4826 (2016). 27229733 10.1093/jxb/erw208

[8] T. Takahashi, T. Suzuki, Y. Osakabe, S. Betsuyaku, Y. Kondo, N. Dohmae, H. Fukuda, K. Yamaguchi-Shinozaki, K. Shinozaki, A small peptide modulates stomatal control via abscisic acid in long-distance signalling. Nature 556, 235–238 (2018). 29618812 10.1038/s41586-018-0009-2

[9] X. Guo, J. Wang, M. Gardner, H. Fukuda, Y. Kondo, J. P. Etchells, X. Wang, M. G. Mitchum, Identification of cyst nematode B-type CLE peptides and modulation of the vascular stem cell pathway for feeding cell formation. PLOS Pathog. 13, e1006142 (2017). 28158306 10.1371/journal.ppat.1006142 PMC5319780

[10] A. Replogle, J. Wang, A. Bleckmann, R. S. Hussey, T. J. Baum, S. Sawa, E. L. Davis, X. Wang, R. Simon, M. G. Mitchum, Nematode CLE signaling in *Arabidopsis* requires CLAVATA2 and CORYNE. Plant J. 65, 430–440 (2011). 21265896 10.1111/j.1365-313X.2010.04433.x

[11] A. Replogle, J. Wang, V. Paolillo, J. Smeda, A. Kinoshita, A. Durbak, F. E. Tax, X. Wang, S. Sawa, M. G. Mitchum, Synergistic interaction of CLAVATA1, CLAVATA2, and receptor-like protein kinase 2 in cyst nematode parasitism of *Arabidopsis*. Mol. Plant Microbe Interact. 26, 87–96 (2013). 22835273 10.1094/MPMI-05-12-0118-FI

[12] J. T. Jones, A. Haegeman, E. G. J. Danchin, H. S. Gaur, J. Helder, M. G. K. Jones, T. Kikuchi, R. Manzanilla-López, J. E. Palomares-Rius, W. M. L. Wesemael, R. N. Perry, Top 10 plant-parasitic nematodes in molecular plant pathology. Mol. Plant Pathol. 14, 946–961 (2013). 23809086 10.1111/mpp.12057 PMC6638764

[13] G. Wang, C. Hu, J. Zhou, Y. Liu, J. Cai, C. Pan, Y. Wang, X. Wu, K. Shi, X. Xia, Y. Zhou, C. H. Foyer, J. Yu, Systemic root-shoot signaling drives jasmonate-based root defense against nematodes. Curr. Biol. 29, 3430–3438.e4 (2019). 31588001 10.1016/j.cub.2019.08.049

[14] Y. L. Yamaguchi, R. Suzuki, J. Cabrera, S. Nakagami, T. Sagara, C. Ejima, R. Sano, Y. Aoki, R. Olmo, T. Kurata, T. Obayashi, T. Demura, T. Ishida, C. Escobar, S. Sawa, Root-knot and cyst nematodes activate procambium-associated genes in *Arabidopsis* roots. Front. Plant Sci. 8, 1195 (2017). 28747918 10.3389/fpls.2017.01195 PMC5506325

[15] J. H. Jun, E. Fiume, J. C. Fletcher, The CLE family of plant polypeptide signaling molecules. Cell. Mol. Life Sci. 65, 743–755 (2008). 18034320 10.1007/s00018-007-7411-5 PMC11131854

[16] J. Kang, X. Wang, T. Ishida, E. Grienenberger, Q. Zheng, J. Wang, Y. Zhang, W. Chen, M. Chen, X.-F. Song, C. Wu, Z. Hu, L. Jia, C. Li, C.-M. Liu, J. C. Fletcher, S. Sawa, G. Wang, A group of CLE peptides regulates de *novo* shoot regeneration in *Arabidopsis thaliana*. New Phytol. 235, 2300–2312 (2022). 35642449 10.1111/nph.18291

[17] S. Nakagami, T. Aoyama, Y. Sato, T. Kajiwara, T. Ishida, S. Sawa, CLE3 and its homologues share overlapping functions in the modulation of lateral root formation through CLV1 and BAM1 in *Arabidopsis thaliana*. Plant J. 6, 1176–1191 (2023). 10.1111/tpj.16103 36628476

[18] N. Czyzewicz, C.-L. Shi, L. D. Vu, B. Van De Cotte, C. Hodgman, M. A. Butenko, I. D. Smet, Modulation of *Arabidopsis* and monocot root architecture by CLAVATA3/EMBRYO SURROUNDING REGION 26 peptide. J. Exp. Bot. 66, 5229–5243 (2015). 26188203 10.1093/jxb/erv360 PMC4526925

[19] J. H. Jun, E. Fiume, A. H. K. Roeder, L. Meng, V. K. Sharma, K. S. Osmont, C. Baker, C. M. Ha, E. M. Meyerowitz, L. J. Feldman, J. C. Fletcher, Comprehensive analysis of *CLE* polypeptide signaling gene expression and overexpression activity in *Arabidopsis*. Plant Physiol. 154, 1721–1736 (2010). 20884811 10.1104/pp.110.163683 PMC2996011

[20] J. Zuo, Q. W. Niu, N. H. Chua, An estrogen receptor-based transactivator XVE mediates highly inducible gene expression in transgenic plants. Plant J. 24, 265–273 (2000). 11069700 10.1046/j.1365-313x.2000.00868.x

[21] A. Kereszt, P. Mergaert, J. Montiel, G. Endre, É. Kondorosi, Impact of plant peptides on symbiotic nodule development and functioning. Front. Plant Sci. 9, 1026 (2018). 30065740 10.3389/fpls.2018.01026 PMC6056668

[22] K. Ohyama, H. Shinohara, M. Ogawa-Ohnishi, Y. Matsubayashi, A glycopeptide regulating stem cell fate in *Arabidopsis thaliana*. Nat. Chem. Biol. 5, 578–580 (2009). 19525968 10.1038/nchembio.182

[23] T. Araya, M. Miyamoto, J. Wibowo, A. Suzuki, S. Kojima, Y. N. Tsuchiya, S. Sawa, H. Fukuda, N. von Wirén, H. Takahashi, CLE-CLAVATA1 peptide-receptor signaling module regulates the expansion of plant root systems in a nitrogen-dependent manner. Proc. Natl. Acad. Sci. U.S.A. 111, 2029–2034 (2014). 24449877 10.1073/pnas.1319953111 PMC3918772

[24] V. K. Sharma, J. C. Fletcher, Maintenance of shoot and floral meristem cell proliferation and fate. Plant Physiol. 129, 31–39 (2002). 12011335 10.1104/pp.010987 PMC1540224

[25] Y. Stahl, R. H. Wink, G. C. Ingram, R. Simon, A signaling module controlling the stem cell niche in *Arabidopsis* root meristems. Curr. Biol. 19, 909–914 (2009). 19398337 10.1016/j.cub.2009.03.060

[26] Y. Stahl, S. Grabowski, A. Bleckmann, R. Kühnemuth, S. Weidtkamp-Peters, K. G. Pinto, G. K. Kirschner, J. B. Schmid, R. H. Wink, A. Hülsewede, S. Felekyan, C. A. M. Seidel, R. Simon, Moderation of *Arabidopsis* Root Stemness by CLAVATA1 and ARABIDOPSIS CRINKLY4 receptor kinase complexes. Curr. Biol. 23, 362–371 (2013). 23394827 10.1016/j.cub.2013.01.045

[27] S. Okamoto, T. Suzuki, M. Kawaguchi, T. Higashiyama, Y. Matsubayashi, A comprehensive strategy for identifying long-distance mobile peptides in xylem sap. Plant J. 84, 611–620 (2015). 26333921 10.1111/tpj.13015

[28] S. Okamoto, A. Kawasaki, Y. Makino, T. Ishida, S. Sawa, Long-distance translocation of CLAVATA3/ESR-related 2 peptide and its positive effect on roots sucrose status. Plant Physiol. 189, 2357–2367 (2022). 35567530 10.1093/plphys/kiac227 PMC9342984

[29] R. Tabata, K. Sumida, T. Yoshii, K. Ohyama, H. Shinohara, Y. Matsubayashi, Perception of root-derived peptides by shoot LRR-RKs mediates systemic N-demand signaling. Science 346, 343–346 (2014). 25324386 10.1126/science.1257800

[30] C. W. Lim, Y. W. Lee, C. H. Hwang, Soybean nodule-enhanced CLE peptides in roots act as signals in GmNARK-mediated nodulation suppression. Plant Cell Physiol. 52, 1613–1627 (2011). 21757457 10.1093/pcp/pcr091

[31] V. Mortier, G. Den Herder, R. Whitford, W. Van de Velde, S. Rombauts, K. D’haeseleer, M. Holsters, S. Goormachtig, CLE peptides control *Medicago truncatula* nodulation locally and systemically. Plant Physiol. 153, 222–237 (2010). 20348212 10.1104/pp.110.153718 PMC2862434

[32] D. E. Reid, B. J. Ferguson, P. M. Gresshoff, Inoculation- and nitrate-induced CLE peptides of soybean control NARK-dependent nodule formation. Mol. Plant Microbe Interact. 24, 606–618 (2011). 21198362 10.1094/MPMI-09-10-0207

[33] D. Ma, S. Endo, S. Betsuyaku, A. Shimotohno, H. Fukuda, CLE2 regulates light-dependent carbohydrate metabolism in *Arabidopsis* shoots. Plant Mol. Biol. 104, 561–574 (2020). 32980951 10.1007/s11103-020-01059-y

[34] M. G. Mitchum, X. Liu, Peptide effectors in phytonematode parasitism and beyond. Annu. Rev. Phytopathol. 60, 97–119 (2022). 35385672 10.1146/annurev-phyto-021621-115932

[35] J. Wang, A. Dhroso, X. Liu, T. J. Baum, R. S. Hussey, E. L. Davis, X. Wang, D. Korkin, M. G. Mitchum, Phytonematode peptide effectors exploit a host post-translational trafficking mechanism to the ER using a novel translocation signal. New Phytol. 229, 563–574 (2021). 32569394 10.1111/nph.16765

[36] G. Huang, R. Dong, R. Allen, E. L. Davis, T. J. Baum, R. S. Hussey, A root-knot nematode secretory peptide functions as a ligand for a plant transcription factor. Mol. Plant Microbe Interact. 19, 463–470 (2006). 16673933 10.1094/MPMI-19-0463

[37] W. B. Rutter, T. Hewezi, T. R. Maier, M. G. Mitchum, E. L. Davis, R. S. Hussey, T. J. Baum, Members of the *Meloidogyne* Avirulence protein family contain multiple plant ligand-like motifs. Phytopathology 104, 879–885 (2014). 25014776 10.1094/PHYTO-11-13-0326-R

[38] G. Huang, R. Allen, E. L. Davis, T. J. Baum, R. S. Hussey, Engineering broad root-knot resistance in transgenic plants by RNAi silencing of a conserved and essential root-knot nematode parasitism gene. Proc. Nati. Acad. Sci. U.S.A. 103, 14302–14306 (2006). 10.1073/pnas.0604698103 PMC1570184 16985000

[39] B. Absmanner, R. Stadler, U. Z. Hammes, Phloem development in nematode-induced feeding sites: The implications of auxin and cytokinin. Front. Plant Sci. 4, 241 (2013). 23847644 10.3389/fpls.2013.00241 PMC3703529

[40] M. Barcala, A. García, J. Cabrera, S. Casson, K. Lindsey, B. Favery, G. García-Casado, R. Solano, C. Fenoll, C. Escobar, Early transcriptomic events in microdissected *Arabidopsis* nematode-induced giant cells. Plant J. 61, 698–712 (2010). 20003167 10.1111/j.1365-313X.2009.04098.x

[41] C. D. Dowd, D. Chronis, Z. S. Radakovic, S. Siddique, T. Schmülling, T. Werner, T. Kakimoto, F. M. W. Grundler, M. G. Mitchum, Divergent expression of cytokinin biosynthesis, signaling and catabolism genes underlying differences in feeding sites induced by cyst and root-knot nematodes. Plant J. 92, 211–228 (2017). 28746737 10.1111/tpj.13647

[42] A. Islam, C. F. Mercer, S. Leung, P. P. Dijkwel, M. T. McManus, Transcription of biotic stress associated genes in white clover (*Trifolium repens L.*) Differs in response to cyst and root-knot nematode infection. PLOS ONE 10, e0137981 (2015). 26393362 10.1371/journal.pone.0137981 PMC4578895

[43] S. J. Shah, M. S. Anjam, B. Mendy, M. A. Anwer, S. S. Habash, J. L. Lozano-Torres, F. M. W. Grundler, S. Siddique, Damage-associated responses of the host contribute to defence against cyst nematodes but not root-knot nematodes. J. Exp. Bot. 68, 5949–5960 (2017). 29053864 10.1093/jxb/erx374 PMC5854129

[44] M. Hayashi-Tsugane, M. Kawaguchi, *Lotus japonicus* HAR1 regulates root morphology locally and systemically under a moderate nitrate condition in the absence of rhizobia. Planta 255, 95 (2022). 35348891 10.1007/s00425-022-03873-8

[45] R. Nishimura, M. Hayashi, G. J. Wu, H. Kouchi, H. Imaizumi-Anraku, Y. Murakami, S. Kawasaki, S. Akao, M. Ohmori, M. Nagasawa, K. Harada, M. Kawaguchi, HAR1 mediates systemic regulation of symbiotic organ development. Nature 420, 426–429 (2002). 12442172 10.1038/nature01231

[46] J. Wopereis, E. Pajuelo, F. B. Dazzo, Q. Jiang, P. M. Gresshoff, F. J. de Bruijn, J. Stougaard, K. Szczyglowski, Short root mutant of *Lotus japonicus* with a dramatically altered symbiotic phenotype. Plant J. 23, 97–114 (2000). 10929105 10.1046/j.1365-313x.2000.00799.x

[47] E. Huault, C. Laffont, J. Wen, K. S. Mysore, P. Ratet, G. Duc, F. Frugier, Local and systemic regulation of plant root system architecture and symbiotic nodulation by a receptor-like kinase. PLOS Genet. 10, e1004891 (2014). 25521478 10.1371/journal.pgen.1004891 PMC4270686

[48] K. Chapman, M. Taleski, H. A. Ogilvie, N. Imin, M. A. Djordjevic, *CEP–CEPR1* signalling inhibits the sucrose-dependent enhancement of lateral root growth. J. Exp. Bot. 70, 3955–3967 (2019). 31056646 10.1093/jxb/erz207 PMC6685651

[49] I. Dimitrov, F. E. Tax, Lateral root growth in *Arabidopsis* is controlled by short and long distance signaling through the LRR RLKs XIP1/CEPR1 and CEPR2. Plant Signal. Behav. 13, e1489667 (2018). 29993313 10.1080/15592324.2018.1489667 PMC6110363

[50] H. Miyazawa, E. Oka-Kira, N. Sato, H. Takahashi, G. J. Wu, S. Sato, M. Hayashi, S. Betsuyaku, M. Nakazono, S. Tabata, K. Harada, S. Sawa, H. Fukuda, M. Kawaguchi, The receptor-like kinase KLAVIER mediates systemic regulation of nodulation and non-symbiotic shoot development in *Lotus japonicus*. Development 137, 4317–4325 (2010). 21098572 10.1242/dev.058891

[51] Z. Luo, J. Lin, Y. Zhu, M. Fu, X. Li, F. Xie, NLP1 reciprocally regulates nitrate inhibition of nodulation through SUNN-CRA2 signaling in *Medicago truncatula*. Plant Commun. 2, 100183 (2021). 34027396 10.1016/j.xplc.2021.100183 PMC8132174

[52] H. Nishida, S. Tanaka, Y. Handa, M. Ito, Y. Sakamoto, S. Matsunaga, S. Betsuyaku, K. Miura, T. Soyano, M. Kawaguchi, T. Suzaki, A NIN-LIKE PROTEIN mediates nitrate-induced control of root nodule symbiosis in *Lotus japonicus*. Nat. Commun. 9, 499 (2018). 29403008 10.1038/s41467-018-02831-x PMC5799372

[53] H. Nishida, S. Nosaki, T. Suzuki, M. Ito, T. Miyakawa, M. Nomoto, Y. Tada, K. Miura, M. Tanokura, M. Kawaguchi, T. Suzaki, Different DNA-binding specificities of NLP and NIN transcription factors underlie nitrate-induced control of root nodulation. Plant Cell 33, 2340–2359 (2021). 33826745 10.1093/plcell/koab103 PMC8364233

[54] W. Dong, Y. Wang, H. Takahashi, CLE-clavata1 signaling pathway modulates lateral root development under sulfur deficiency. Plan. Theory 8, 103 (2019). 10.3390/plants8040103 PMC6524044 31003469

[55] D. Zhao, Y. You, H. Han, X. Zhu, Y. Wang, Y. Duan, Y. Xuan, L. Chen, The role of sugar transporter genes during early infection by root-knot nematodes. Int. J. Mol. Sci. 19, 302 (2018). 29351253 10.3390/ijms19010302 PMC5796247

[56] A. Martínez-Medina, C. M. Mbaluto, A. Maedicke, A. Weinhold, F. Vergara, N. M. van Dam, Leaf herbivory counteracts nematode-triggered repression of jasmonate-related defenses in tomato roots. Plant Physiol. 187, 1762–1778 (2021). 34618073 10.1093/plphys/kiab368 PMC8566281

[57] B. J. De Young, K. L. Bickle, K. J. Schrage, P. Muskett, K. Patel, S. E. Clark, The CLAVATA1-related BAM1, BAM2 and BAM3 receptor kinase-like proteins are required for meristem function in *Arabidopsis*. Plant J. 45, 1–16 (2006). 16367950 10.1111/j.1365-313X.2005.02592.x

[58] K. Fisher, S. Turner, PXY, a receptor-like kinase essential for maintaining polarity during plant vascular-tissue development. Curr. Biol. 17, 1061–1066 (2007). 17570668 10.1016/j.cub.2007.05.049

[59] A. Kinoshita, S. Betsuyaku, Y. Osakabe, S. Mizuno, S. Nagawa, Y. Stahl, R. Simon, K. Yamaguchi-Shinozaki, H. Fukuda, S. Sawa, RPK2 is an essential receptor-like kinase that transmits the CLV3 signal in *Arabidopsis*. Development 137, 4327 (2010). 10.1242/dev.048199 20978082

[60] Y. L. Yamaguchi, T. Ishida, M. Yoshimura, Y. Imamura, C. Shimaoka, S. Sawa, A collection of mutants for CLE-peptide-encoding genes in *Arabidopsis* generated by CRISPR/Cas9-mediated gene targeting. Plant Cell Physiol. 58, 1848–1856 (2017). 29036337 10.1093/pcp/pcx139

[61] A. Imoto, M. Yamada, T. Sakamoto, A. Okuyama, T. Ishida, S. Sawa, M. Aida, A ClearSee-based clearing protocol for 3D visualization of *Arabidopsis thaliana* embryos. Plan. Theory 10, 190 (2021). 10.3390/plants10020190 PMC7909245 33498275

[62] J. D. Pédelacq, S. Cabantous, T. Tran, T. C. Terwilliger, G. S. Waldo, Engineering and characterization of a superfolder green fluorescent protein. Nat. Biotechnol. 24, 79–88 (2006). 16369541 10.1038/nbt1172

[63] D. S. Favero, A. Kawamura, M. Shibata, A. Takebayashi, J.-H. Jung, T. Suzuki, K. E. Jaeger, T. Ishida, A. Iwase, P. A. Wigge, M. M. Neff, K. Sugimoto, AT-hook transcription factors restrict petiole growth by antagonizing PIFs. Curr. Biol. 30, 1454–1466.e6 (2020). 32197081 10.1016/j.cub.2020.02.017

[64] H. Nishiyama, B. T. Ngan, S. Nakagami, C. Ejima, T. Ishida, S. Sawa, Protocol for root-knot nematode culture by a hydroponic system and nematode inoculation to *Arabidopsi*. Japanese J. Nematol. 45, 45–49 (2015).

[65] C. G. N. Turnbull, J. P. Booker, H. M. O. Leyser, Micrografting techniques for testing long-distance signalling in *Arabidopsis*. Plant J. 32, 255–262 (2002). 12383090 10.1046/j.1365-313x.2002.01419.x

[66] D. Gaidatzis, A. Lerch, F. Hahne, M. B. Stadler, QuasR: Quantification and annotation of short reads in R. Bioinformatics 31, 1130–1132 (2015). 25417205 10.1093/bioinformatics/btu781 PMC4382904

[67] J. Sun, T. Nishiyama, K. Shimizu, K. Kadota, TCC: An R package for comparing tag count data with robust normalization strategies. BMC Bioinformatics 14, 219 (2013). 23837715 10.1186/1471-2105-14-219 PMC3716788

[68] T. Kondo, T. Nakamura, K. Yokomine, Y. Sakagami, Dual assay for MCLV3 activity reveals structure—Activity relationship of CLE peptides. Biochem. Biophys. Res. Commun. 377, 312–316 (2008). 18848920 10.1016/j.bbrc.2008.09.139

[69] H. Shinohara, M. Ogawa, Y. Sakagami, Y. Matsubayashi, Identification of ligand binding site of phytosulfokine receptor by on-column photoaffinity labeling. J. Biol. Chem. 282, 124–131 (2007). 17092941 10.1074/jbc.M604558200

[70] R. Wang, K. Guegler, S. T. LaBrie, N. M. Crawford, Genomic analysis of a nutrient response in *Arabidopsis* reveals diverse expression patterns and novel metabolic and potential regulatory genes induced by nitrate. Plant Cell 12, 1491–1509 (2000). 10948265 10.1105/tpc.12.8.1491 PMC149118

