# Supplementary material for: Root-knot nematode modulates plant CLE3-CLV1 signaling as a long-distance signal for successful infection
Source: Sci Adv. 2023 Jun 2;9(22):eadf4803. doi: 10.1126/sciadv.adf4803 (PMC10413670; doi:10.1126/sciadv.adf4803)
Supplement: Supplementary file 1 — Figs. S1 to S9 Tables S1 to S3 References [file sciadv.adf4803_sm.pdf]

Science Advances
AAAS

Supplementary Materials for

Root-knot nematode modulates plant CLE3-CLV1 signaling as a long-distance signal for successful infection

Satoru Nakagami *et al.*

Corresponding author: Shinichiro Sawa, sawa@kumamoto-u.ac.jp

Sci. Adv. **9**, eadf4803 (2023)
DOI: 10.1126/sciadv.adf4803

This PDF file includes:

Figs. S1 to S9
Tables S1 to S3
References

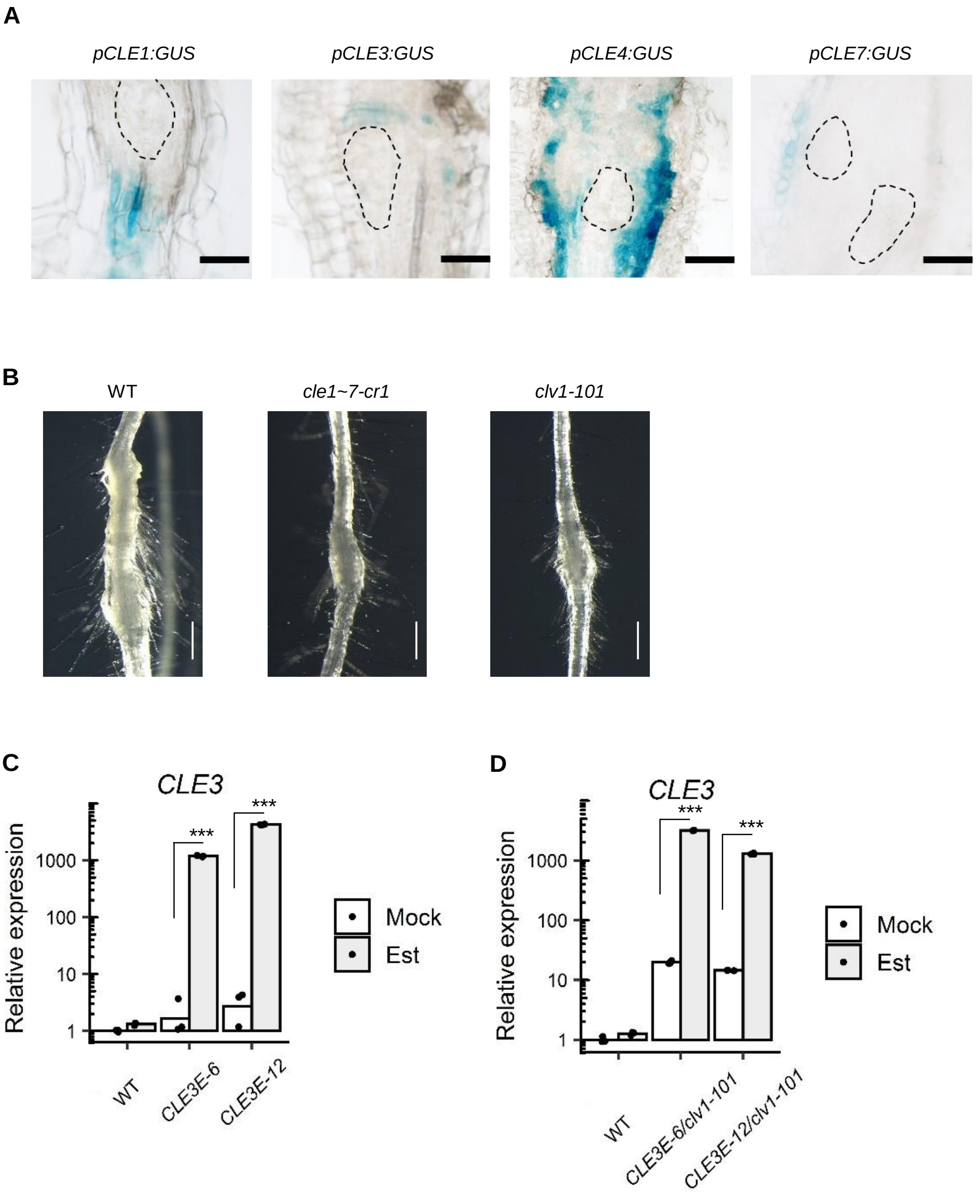

Figure S1. Histochemical analysis and *CLE3* expression in *CLE3*-inducible transgenic plants.
(A) GUS-stained longitudinal sections of *pCLE1:GUS*, *pCLE3:GUS*, *pCLE4:GUS* and *pCLE7:GUS* transgenic plants 5 dpi galls (6 individuals were observed with similar results). Bars = 50 µm. Broken lines denote giant cells. (B) Representative images of 7 dpi galls formed on a primary root in wild type, *cle1~7-cr1* and *clv1-101*. Bars = 200 µm. (C and D) Expression levels of *CLE3* in roots of wild type, *CLE3E-6* and *CLE3E-12* (C) and wild type, *CLE3E-6/clv1-101* and *CLE3E-12/clv1-101* (D) plants that were mock-treated or treated with 10 µM β-estradiol for 18 hours (n = 3 biological replicates). Experiment was repeated three times with similar results. ***P < 0.001 by Student's t-test.

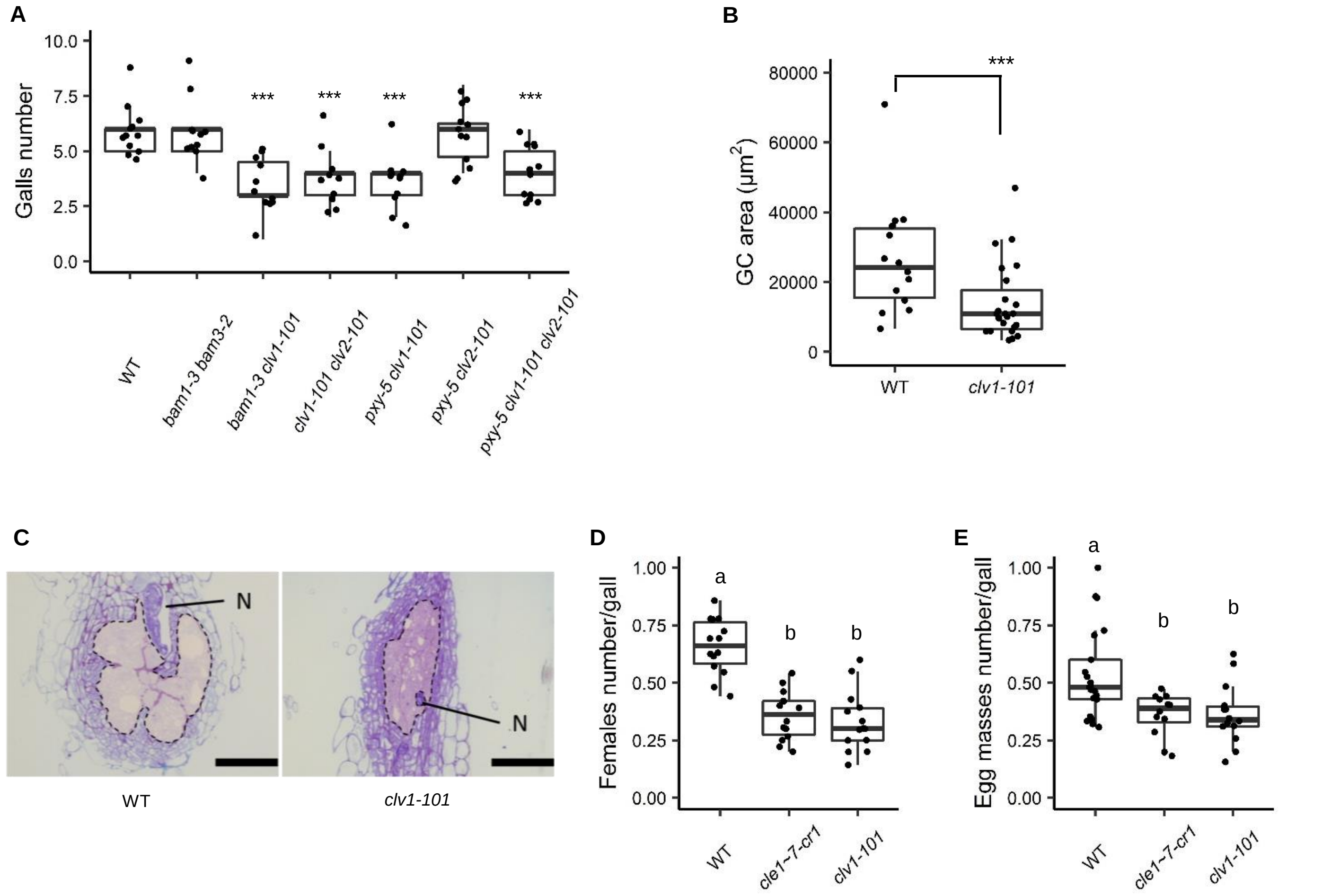

Figure S2. RKN infection assay using LRR mutants.

(A) Gall numbers per plant in wild type, *bam1-3 bam3-2*, *bam1-3 clv1-101*, *clv1-101 clv2-101*, *pxy-5 clv1-101*, *pxy-5 clv2-101* and *pxy-5 clv1-101 clv2-101* at 7 dpi ($n \geq 12$). Asterisks denote significant differences (***$P < 0.001$, one-way ANOVA followed by Dunnett's multiple test when compared to wild type). (B) GC areas of 7 dpi galls in wild type and *clv1-101* ($n \geq 14$). ***$P < 0.001$ by Student's *t*-test. (C) Representative wild type and *clv1-101* 7 dpi gall section images from (B). Bars = 100 μm. N, nematode. Broken lines, GCs. (D) Mature female numbers per gall in wild type, *cle1~7* and *clv1-101* at 28 dpi ($n = 14$). (E) Egg mass numbers per gall in wild type, *cle1~7-cr1* and *clv1-101* at 42 dpi ($n \geq 12$). Alphabets denote significant differences ($P < 0.05$, one-way ANOVA followed by Tukey's test).

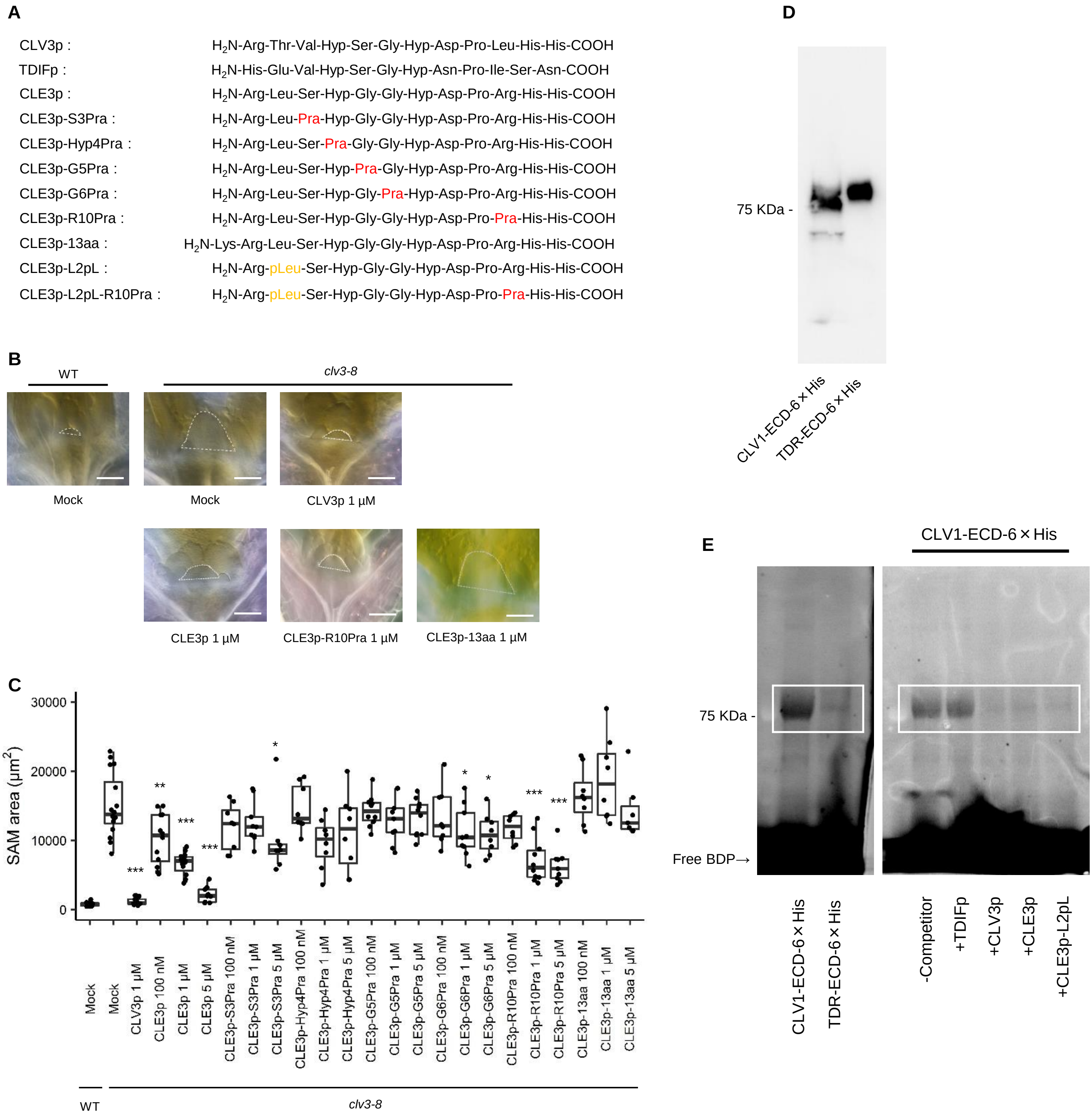

Figure S3. Design of the labelled CLE3 ligand for photoaffinity binding assay.
(A) Amino acid sequences of synthetic CLE3 peptide ligand candidates for the photoffinity binding assay. Pra: propargylglycine (red). pLeu: photo-leucine (yellow). Hyp: hydroxyprorine. (B) Representative micrographs of the SAMs of wild type and *clv3-8* seedlings treated with the CLE3 peptide ligand candidates. Broken lines denote SAM. Bars = 100 µm. (C) Quantified SAM areas at 7 days after peptide treatment ($n \geq 7$). Asterisks denote significant differences (*$P < 0.05$, **$P < 0.01$, ***$P < 0.001$, one-way ANOVA followed by Dunnett's multiple test when compared to Mock treated *clv3-8*). (D) Anti-His tag western blot of total proteins contained in the supernatants of the transfected Sf9 insect cells culture. (E) Rectangles delimit cropped areas used in the indicated panels in Figure 2E (left panel) and F (right panel).

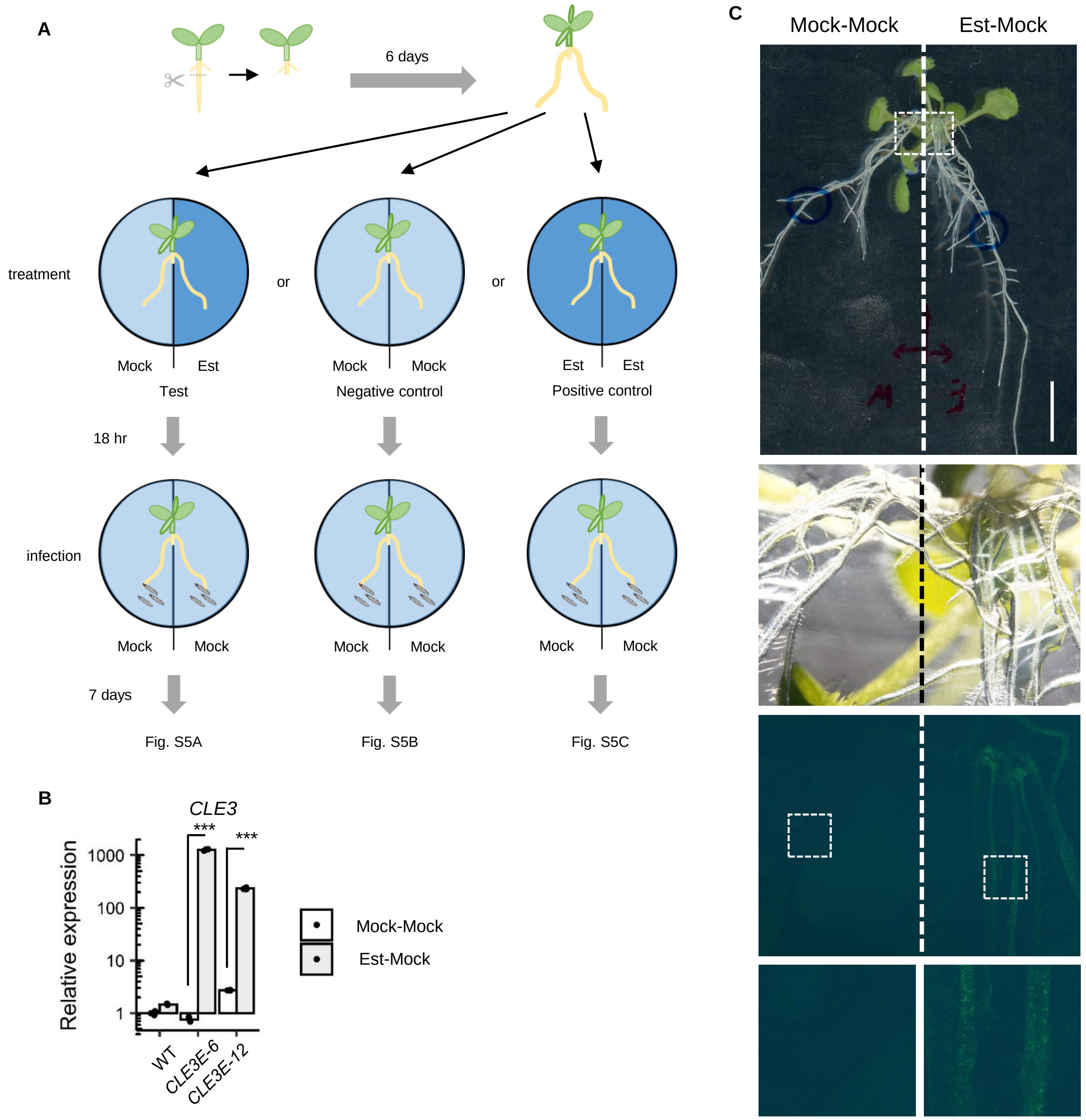

A
6 days
treatment
or
or
Mock
Est
Test
Mock
Mock
Negative control
Est
Est
Positive control
18 hr
infection
Mock
Mock
Mock
Mock
Mock
Mock
7 days
Fig. S5A
Fig. S5B
Fig. S5C
B
CLE3
***
***
Relative expression
1000
100
10
1
WT
CLE3E-6
CLE3E-12
Mock-Mock
Est-Mock
C
Mock-Mock
Est-Mock

Figure S4. Experimental design for the infection assay with split-root culture system.

(A) Schematics illustrating the experimental design for the infection assay with split-root culture system. Vertically grown 7-day-old seedlings had their primary roots cut off to induce lateral roots development for an additional 6 days. Then, seedlings with healthy lateral root growths were transferred onto two-compartments petri dishes containing Mock/Mock, Est/Est or Mock/Est media. After 18 hours, all seedlings were transferred onto Mock/Mock media to terminate the estradiol induction, and then inoculated with approximately 80 RKN per compartment. (B) Expression levels of *CLE3* in roots of wild type, *CLE3E-6* and *CLE3E-12* plants under the test treatment (Mock-Mock and Est-Mock) with split-root culture system ($n = 3$ biological replicates). Total RNA was extracted from roots grown on the Mock/Est media for 18 hours and then grown on the Mock/Mock media for 2 days. Experiment was repeated twice with similar results. ***$P < 0.001$ by Student's *t*-test. (C) Expression pattern of H2B-sfGFP in the transgenic plant expressing the *H2B-sfGFP* under the control of the β-estradiol-inducible promoter (designated as *pER8-GW-H2B-sfGFP* plant) with split-root culture system. The H2B-sfGFP signal was observed in roots grown on the Mock/Est media for 18 hours and then grown on the Mock/Mock media for 2 days. Top row: Bright-field image of the *pER8-GW-H2B-sfGFP* plant. Second row from the top: Magnified image of the highlighted region of the top row. Second row from the bottom: Detection of GFP signal. Bottom row: Magnified images of the highlighted regions of the second row from the bottom.

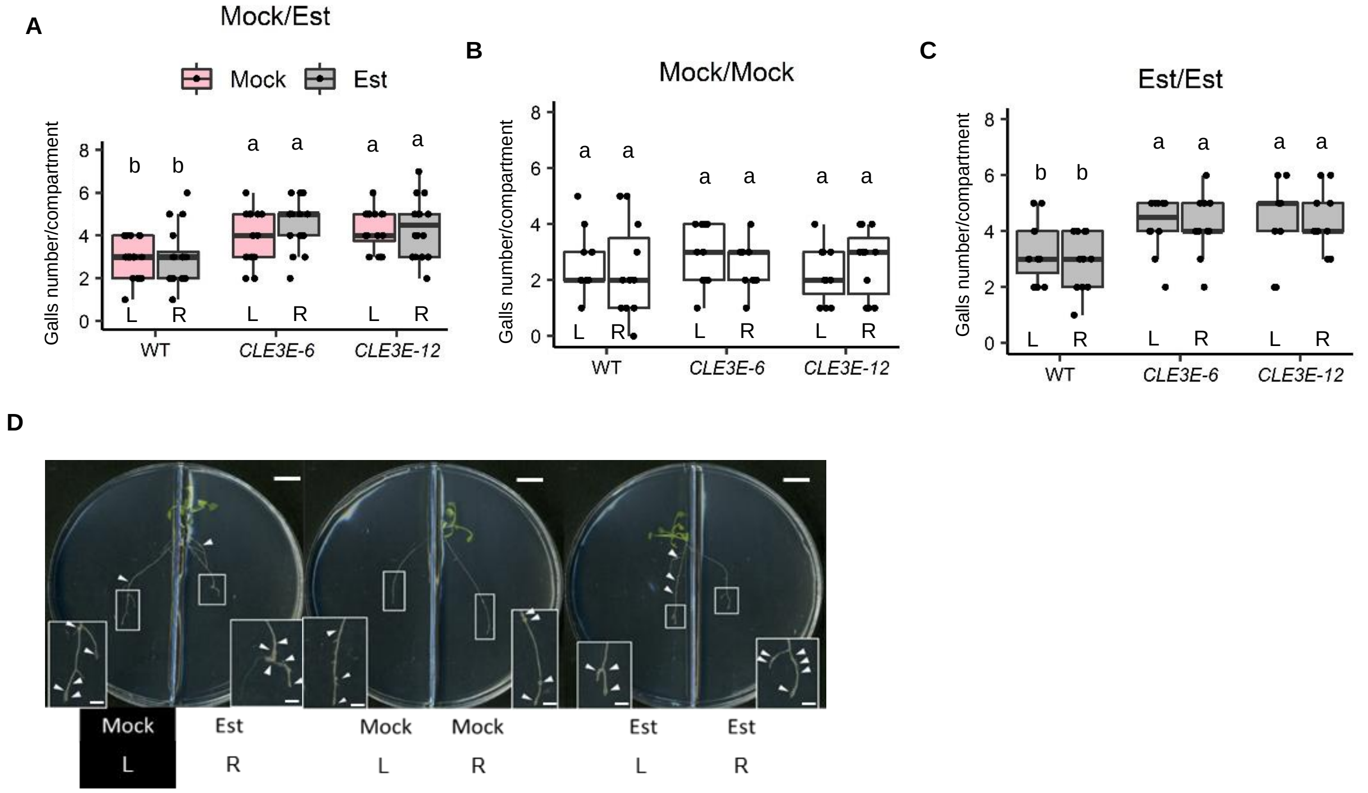

Figure S5. CLE3-mediated gall-formation signal disperses systemically.
(A-C) Gall numbers per compartment in wild type, *CLE3E-6* and *CLE3E-12* plants at 7 dpi using split-root culture system with mock-treatment in the left compartment plus 10 µM β-estradiol on the right compartment (A), mock treatment on both sides (B), and 10 µM β-estradiol on both sides (C) (see also Table S2). Sample sizes are listed in Table S2. Alphabets denote significant differences ($P < 0.05$, two-way ANOVA followed by Tukey's test). (D) Representative images of *CLE3E-6* seedling grown in the split-root culture system at 7 dpi (scale bar = 1 mm). Insets show magnified images of the highlighted root regions (scale bar = 200 µm), galls are marked with arrowhead. L, left compartment. R, right compartment.

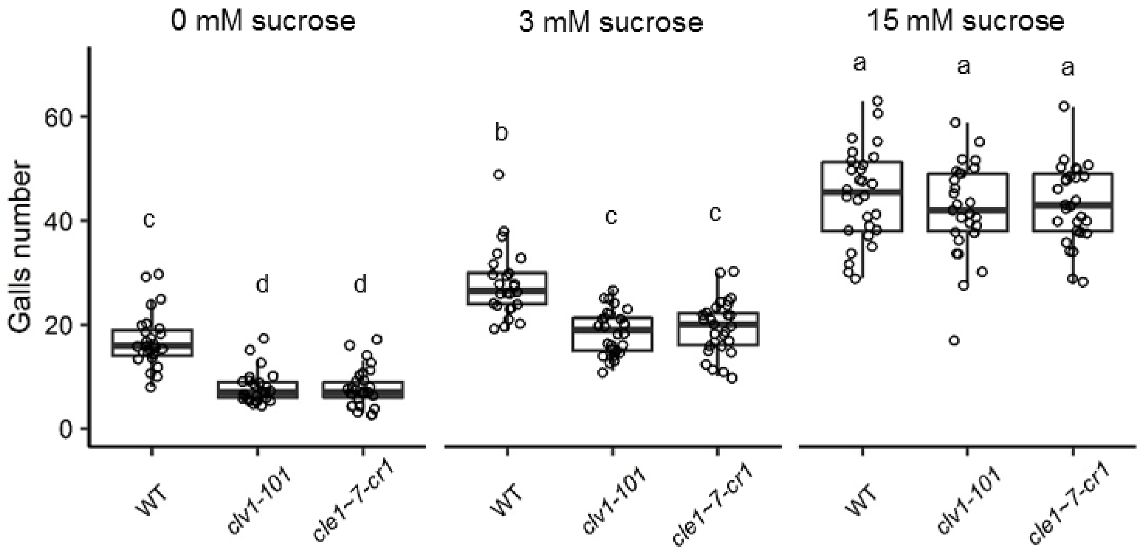

Figure S6. Sucrose rescues cle*1~7-cr1* and *clv1-101* gall formation defects.
Gall numbers in the wild type, *clv1-101* and *cle1~7-cr1* at 7 dpi under 0 mM, 3 mM and 15 mM sucrose ($n \geq 26$). Alphabets denote significant difference (two-way ANOVA followed by Tukey's test).

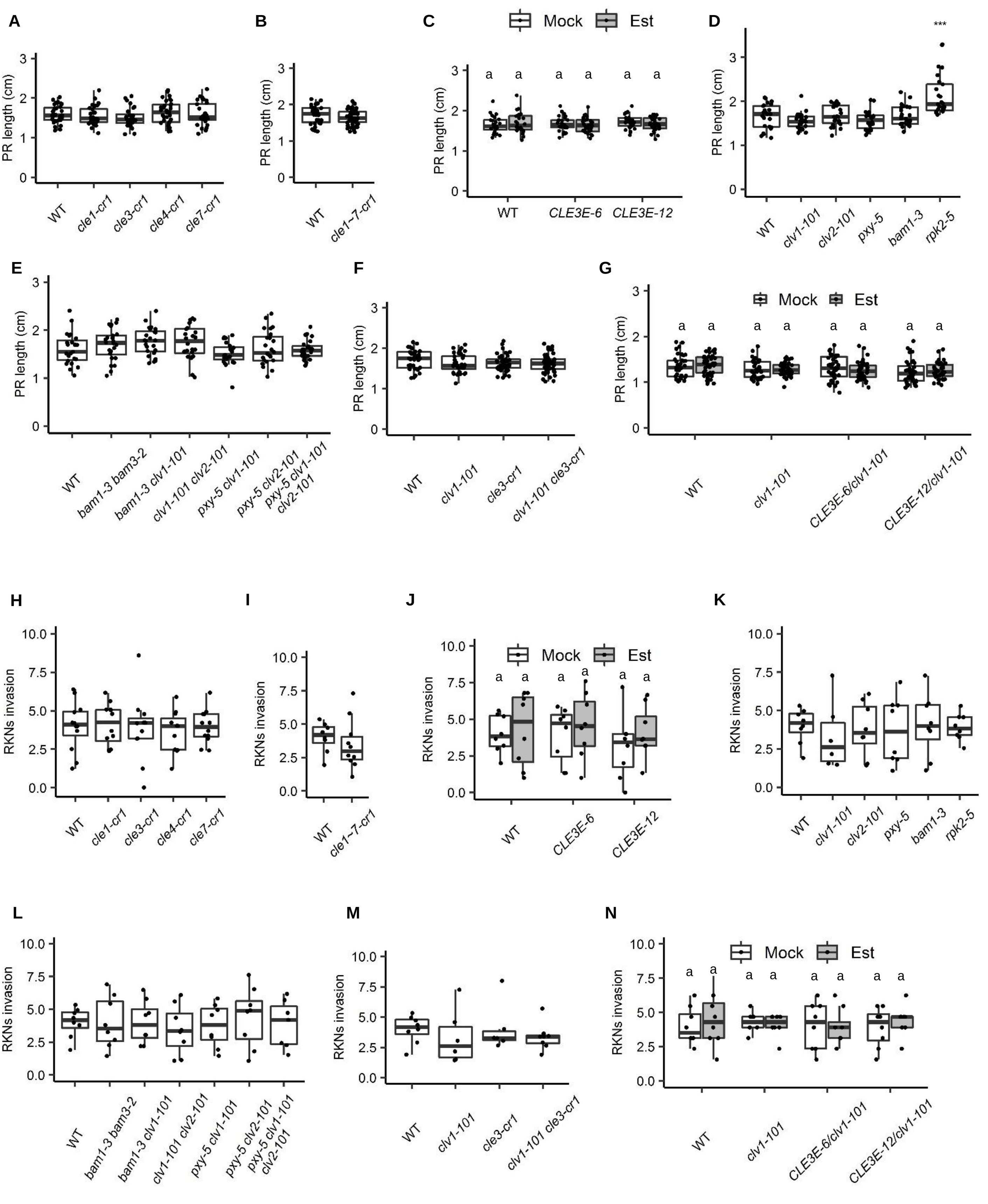
A
B
C
D
Mock
Est
***
PR length (cm)
0
1
2
3
WT
cle1-cr1
cle3-cr1
cle4-cr1
cle7-cr1
cle1~7-cr1
CLE3E-6
CLE3E-12
clv1-101
clv2-101
pxy-5
bam1-3
rpk2-5
a
E
F
G
bam1-3 bam3-2
bam1-3 clv1-101
clv1-101 clv2-101
pxy-5 clv1-101
pxy-5 clv2-101
pxy-5 clv1-101 clv2-101
cle3-cr1
clv1-101 cle3-cr1
CLE3E-6/clv1-101
CLE3E-12/clv1-101
H
I
J
K
RKNs invasion
0.0
2.5
5.0
7.5
10.0
L
M
N

Figure S7. CLEs and CLV1 are not involved in root growth and RKN invasion response.
(A-G) Primary root (PR) length of the seven-day-old *cle1-cr1*, *cle3-cr1*, *cle4-cr1* and *cle7-cr1* seedlings (n = 32, related to Figure 1C) (A), *cle1~7-cr1* seedlings ($n \geq 36$, related to Figure 1D) (B), *CLE3E-6* and *CLE3E-12* seedlings grown on MS plates with mock or 10 μM β-estradiol for 18 hours ($n \geq 24$, related to Figure 1E) (C), *clv1-101*, *clv2-101*, *pxy-5*, *bam1-3* and *rpk2-5* seedlings ($n \geq 24$, related to Figure 2A) (D), *bam1-3 bam3-2*, *bam1-3 clv1-101*, *clv1-101 clv2-101*, *pxy-5 clv1-101*, *pxy-5 clv2-101* and *pxy-5 clv1-101 clv2-101* seedlings ($n \geq$ 23, related to Figure S2A) (E), *clv1-101*, *cle3-cr1* and *clv1-101 cle3-cr1* seedlings ($n \geq 36$, related to Figure 2C) (F), *clv1-101*, *CLE3E-6/clv1-101* and *CLE3E-12/clv1-101* seedlings grown on MS plates with mock or 10 μM β-estradiol for 18 hours (n = 40, related to Figure 2D) (G). (H-N) Invaded RKN numbers per seedling at 3 dpi in *cle1-cr1*, *cle3-cr1*, *cle4-cr1* and *cle7-cr1* ($n \geq 11$, related to Figure 1C). Roots of RKN inoculated seedlings were stained with acid fuchsin and then stained RKNs inside the roots were counted. (H), *cle1~7-cr1* mutants ($n \geq 8$, related to Figure 1D and Figure 2B) (I), *CLE3E-6* and *CLE3E-12* treated with mock or 10 μM β-estradiol for 18 hours (n = 8, related to Figure 1E) (J), *clv1-101*, *clv2-101*, *pxy-5*, *bam1-3* and *rpk2-5* ($n \geq 6$, related to Figure 2A) (K), *bam1-3 bam3-2*, *bam1-3 clv1-101*, *clv1-101 clv2-101*, *pxy-5 clv1-101*, *pxy-5 clv2-101* and *pxy-5 clv1-101 clv2-101* (n = 8, related to Figure S3) (L), *clv1-101*, *cle3-cr1* and *clv1-101 cle3-cr1* ($n \geq 6$, related to Figure 2C) (M), *clv1-101*, *CLE3E-6/clv1-101* and *CLE3E-12/clv1-101* treated with mock or 10 μM β-estradiol for 18 hours (n ≥ 8, *CLE3E-12/clv1-101*, related to Figure 2D) (N). Asterisk denotes significant difference (***$P < 0.001$, one-way ANOVA followed by Dunnett's multiple test when compared to wild type) (A, B, D-F, H, I and K-M). Alphabets denote significant differences ($P < 0.05$, two-way ANOVA followed by Tukey's test) (C, G, J and N).

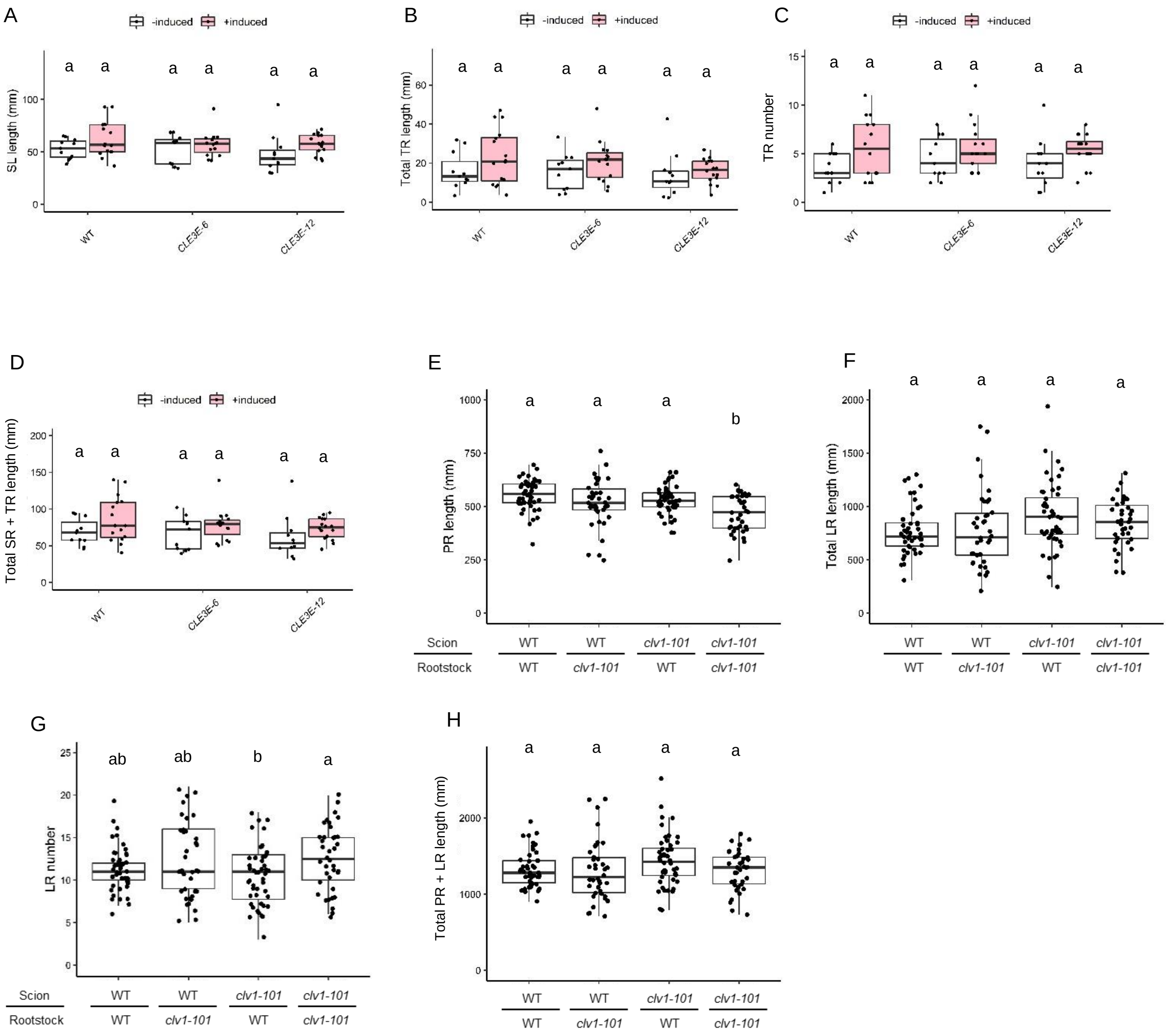

Figure S8. CLE3 overexpression and CLV1 defect do not affect general root growth.
(A-D) Secondary root (SL) length (A), total tertiary root (TR) length (B), TR number (C) and total SL + TR length (D) of plants used in the split-root experiments (related to Figure 4B). Sample sizes are listed in Table S2. Alphabets denote significant difference ($P < 0.05$, two-way ANOVA followed by Tukey's test). (E-H) Primary root (PR) length (E), total lateral root (LR) length (F), LR number (G) and total PR + LR length (H) of the grafted chimeric plants ($n \geq 38$, related to Figure 4C). Alphabets denote significant difference ($P < 0.05$, one-way ANOVA followed by Tukey's test).

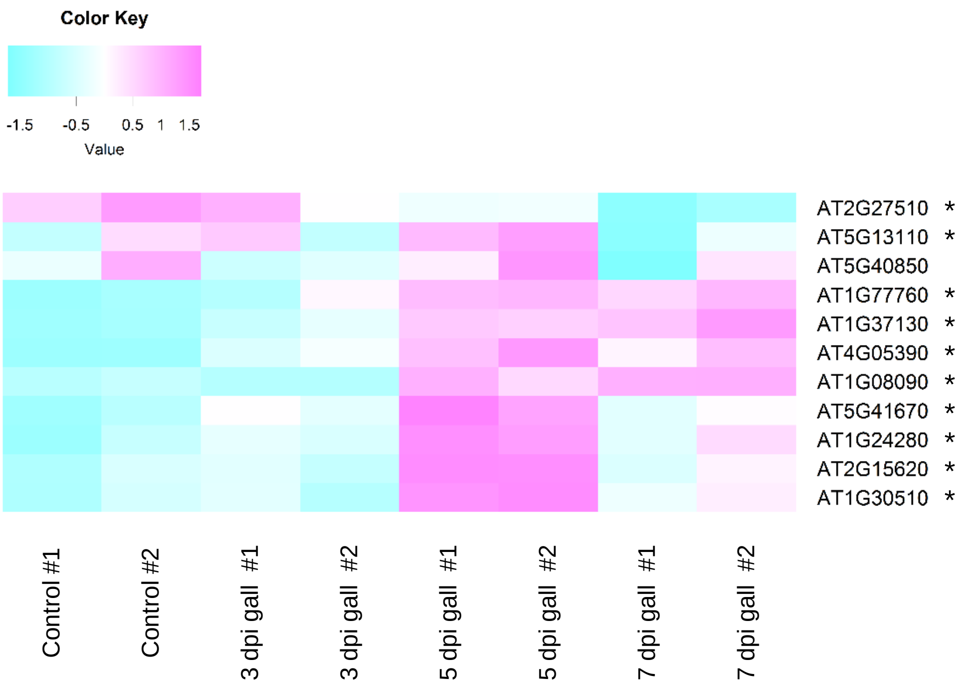

Figure S9. Expression profiles of nitrate-responsive genes in RKN-induced galls.

Heat map depicting the differentially expressed genes in 3 dpi, 5 dpi and 7 dpi galls. Expression data were analyzed by Yamaguchi et al. (2017) (14). The source data can be found at DDBJ (accession number: PRJDB5797), https://ddbj.nig.ac.jp/search/?query=%22PRJDB5797%22. 10 out of the 11 nitrate-responsive genes showed significantly difference in expression levels in galls. The list of nitrate-responsive genes was obtained from Wang et al. (2000) (70). Asterisks denote significant difference (FDR < 0.05).

Table S1. List of *CLE* genes expressed in wild type roots and/or galls.

Gene ID	Gene name	3 dpi	5 dpi	7 dpi
AT1G73165	*CLE1*	0.770	-0.146	-0.473
AT4G18510	*CLE2*	0.101	-1.439	-1.761
**AT1G06225**	***CLE3***	**3.415**	**5.076**	**5.723**
AT2G31081	*CLE4*	2.907	0.750	1.997
AT2G31083	*CLE5*	0.756	-3.678	-1.825
AT2G31085	*CLE6*	2.430	0.758	1.030
AT2G31082	*CLE7*	1.868	-0.716	0.912
AT1G49005	*CLE11*	2.429	0.026	-1.308
AT1G68795	*CLE12*	-0.983	-0.779	-0.894
AT1G73965	*CLE13*	1.542	1.217	0.357
AT1G63245	*CLE14*	0.108	-0.130	-0.596
AT2G01505	*CLE16*	0.317	-0.972	-1.949
AT1G70895	*CLE17*	0.260	0.343	-0.091
AT1G05065	*CLE20*	1.650	2.490	2.589
AT5G12235	*CLE22*	-2.426	-0.497	-0.498
AT3G28455	*CLE25*	-0.007	-0.026	0.665
AT1G69970	*CLE26*	-1.567	-1.817	-1.322
AT3G25905	*CLE27*	0.488	-0.218	-3.439
AT3G24770	*CLE41*	-0.770	0.402	0.501
AT2G34925	*CLE42*	-0.901	-0.938	-0.192
AT1G25425	*CLE43*	2.951	1.924	1.482
AT4G13195	*CLE44*	1.546	3.028	2.845
AT1G69588	*CLE45*	-0.760	-0.374	0.561

Expression data were originally analyzed by Yamaguchi et al. (2017) (Data ref: Yamaguchi et al., 2017) (14). Relative gene expression are shown as log2 fold changes (FC) in 3 dpi, 5 dpi and 7 dpi galls in wild type, normalized to transcript levels in un-inoculated roots. Transcripts for 23 out of the 32 Arabidopsis *CLE* genes were detected in un-inoculated roots and galls from at least one of these time points. Bolded font denotes the expression levels of *CLE3*, which was the most highly up-regulated gene in galls.

Table S2. RKN infection assay using the split-root culture system.

Genotype	Compartment	The number of galls/comp.	The number of samples
Wild type	Mock/Mock (L)	2.64 ± 1.12	n = 11
	Mock/Mock (R)	2.36 ± 1.43	n = 11
	Est/Est (L)	3.27 ± 1.1	n = 11
	Est/Est (R)	2.91 ± 1.04	n = 11
	Mock (opposite side: Est)	2.94 ± 0.93	n = 16
	Est (opposite side: Mock)	3.06 ± 1.34	n = 16
CLE3E-6	Mock/Mock (L)	2.82 ± 1.08	n = 11
	Mock/Mock (R)	2.55 ± 0.82	n = 11
	Est/Est (L)	4.2 ± 1.03	n = 10
	Est/Est (R)	4.18 ± 1.08	n = 11
	Mock (opposite side: Est)	3.87 ± 1.25	n = 15
	Est (opposite side: Mock)	4.5 ± 1.22	n = 14
CLE3E-12	Mock/Mock (L)	2.27 ± 1.01	n = 11
	Mock/Mock (R)	2.64 ± 1.21	n = 11
	Est/Est (L)	4.4 ± 1.43	n = 10
	Est/Est (R)	4.4 ± 1.07	n = 10
	Mock (opposite side: Est)	4.25 ± 0.93	n = 16
	Est (opposite side: Mock)	4.38 ± 1.36	n = 16

Table S3. List of primers for qRT-PCR and vector construction.

Primer name	Direction	Sequence 5'-3'	Reference
CLE1-qRT	F	GTCTCGACCGATGTTCCCA	
	R	CCAGGACTGAGTCTCATGGACT	
CLE2-qRT	F	TATCCTCAATCGCCGCTG	
	R	ACCGCTCTGGAGATTTCCC	
CLE3-qRT	F	CCTGCTTCTAGTACTCGAATTGAC	
	R	ATTTCCAAGGATCGTCTCTTCG	
CLE4-qRT	F	GCCTTATATTGCTTCTACTCGAGTTC	
	R	AAGGGTGCCTAGAACCGTTTG	
CLE5-qRT	F	CGACTTTGATCCTCAAGCAA	
	R	ACTATCCATATTGCCCATGGA	
CLE6-qRT	F	CAAGCTCGAATCCTCCGTAC	
	R	CGTCTCTCGTCTTGACCTTTG	
CLE7-qRT	F	AATTGAAATGGAAGGGAGGATAC	
	R	CTCTCGGCCAATACGCTTTAG	
GAPDH-qRT	F	TTAGTCGCAACCTGAAGCCATC	
	R	TTCCACTGCTACTTGACCTTCG	
TUB4-qRT	F	CACCGAAGGTGCTGAGTTGATT	
	R	GTTCCTCCTCCCAATGAATGACAC	
CLE3-ORF	F	CGACTCTAGCCTCGAATGGCAAGTCTCAAGTTATGGG	
	R	GCCTGGATCGACTAGTCAGTGATGCCTCGGGTC	
CLV1ECD-6×His-stop-F	F	actgcagaaactcccATGGCGATGAGACTTTTG	
CLV1ECD-6×His-stop-R	R	TAAGATCTGGTACCCCTAGTGGTGATGGTGATGATGGGAGGTTTGTCCTGGCCGTG	
TDR-ECD-6xHis-pPSC8-F	F	actgcagaaactcccATGAAAAAGAAGAACATTTCTCCTTC	
TDR-ECD-6xHis-pPSC8_R1	R	CTAGTGGTGATGGTGATGATGATCAGAATTGCAAGGTTTT	
TDR-ECD-6xHis-pPSC8_R2	R	TAAGATCTGGTACCCCTAGTGGTGATGGTGATGATG	
H2B_entry_F	F	TGAAACCCAGCTTTCTTGTACAAAGTTGGC	Imoto et al., (2021) (61)
H2B_entry_R	R	ACCGCCAGATCCCCCAGAACTCGTAAACTTCG	Imoto et al., (2021) (61)
mScarlet_F	F	gggggatctggcggtATGGTGAGCAAGGGCGAGGC	Imoto et al., (2021) (61)
sfGFP-R	R	gaaagctgggttTCACTTGTACAGCTCGTCCATG	

REFERENCES AND NOTES

1. G. Pearce, D. Strydom, S. Johnson, C. A. Ryan, A polypeptide from tomato leaves induces wound-inducible proteinase inhibitor proteins. *Science* **253**, 895–897 (1991).

2. S. Okamoto, E. Ohnishi, S. Sato, H. Takahashi, M. Nakazono, S. Tabata, M. Kawaguchi, Nod factor/nitrate-induced *CLE* genes that drive HAR1-mediated systemic regulation of nodulation. *Plant Cell Physiol.* **50**, 67–77 (2009).

3. S. Okamoto, H. Shinohara, T. Mori, Y. Matsubayashi, M. Kawaguchi, Root-derived CLE glycopeptides control nodulation by direct binding to HAR1 receptor kinase. *Nat. Commun.* **4**, 2191 (2013).

4. J. M. Cock, S. McCormick, A large family of genes that share homology with *CLAVATA3*. *Plant Physiol.* **126**, 939–942 (2001).

5. K. Oelkers, N. Goffard, G. F. Weiller, P. M. Gresshoff, U. Mathesius, T. Frickey, Bioinformatic analysis of the CLE signaling peptide family. *BMC Plant Biol.* **8**, 1 (2008).

6. Y. Ito, I. Nakanomyo, H. Motose, K. Iwamoto, S. Sawa, N. Dohmae, H. Fukuda, Dodeca-CLE peptides as suppressors of plant stem cell differentiation. *Science* **313**, 842–845 (2006).

7. L. Y. Yamaguchi, T. Ishida, S. Sawa, CLE peptides and their signaling pathways in plant development. *J. Exp. Bot.* **67**, 4813–4826 (2016).

8. T. Takahashi, T. Suzuki, Y. Osakabe, S. Betsuyaku, Y. Kondo, N. Dohmae, H. Fukuda, K. Yamaguchi-Shinozaki, K. Shinozaki, A small peptide modulates stomatal control via abscisic acid in long-distance signalling. *Nature* **556**, 235–238 (2018).

9. X. Guo, J. Wang, M. Gardner, H. Fukuda, Y. Kondo, J. P. Etchells, X. Wang, M. G. Mitchum, Identification of cyst nematode B-type CLE peptides and modulation of the vascular stem cell pathway for feeding cell formation. *PLOS Pathog.* **13**, e1006142 (2017).

10. A. Replogle, J. Wang, A. Bleckmann, R. S. Hussey, T. J. Baum, S. Sawa, E. L. Davis, X. Wang, R. Simon, M. G. Mitchum, Nematode CLE signaling in *Arabidopsis* requires CLAVATA2 and CORYNE. *Plant J.* **65**, 430–440 (2011).

11. A. Replogle, J. Wang, V. Paolillo, J. Smeda, A. Kinoshita, A. Durbak, F. E. Tax, X. Wang, S. Sawa, M. G. Mitchum, Synergistic interaction of CLAVATA1, CLAVATA2, and receptor-like protein kinase 2 in cyst nematode parasitism of *Arabidopsis*. *Mol. Plant Microbe Interact.* **26**, 87–96 (2013).

12. J. T. Jones, A. Haegeman, E. G. J. Danchin, H. S. Gaur, J. Helder, M. G. K. Jones, T. Kikuchi, R. Manzanilla-López, J. E. Palomares-Rius, W. M. L. Wesemael, R. N. Perry, Top 10 plant-parasitic nematodes in molecular plant pathology. *Mol. Plant Pathol.* **14**, 946–961 (2013).

13. G. Wang, C. Hu, J. Zhou, Y. Liu, J. Cai, C. Pan, Y. Wang, X. Wu, K. Shi, X. Xia, Y. Zhou, C. H. Foyer, J. Yu, Systemic root-shoot signaling drives jasmonate-based root defense against nematodes. *Curr. Biol.* **29**, 3430–3438.e4 (2019).

14. Y. L. Yamaguchi, R. Suzuki, J. Cabrera, S. Nakagami, T. Sagara, C. Ejima, R. Sano, Y. Aoki, R. Olmo, T. Kurata, T. Obayashi, T. Demura, T. Ishida, C. Escobar, S. Sawa, Root-knot and cyst nematodes activate procambium-associated genes in *Arabidopsis* roots. *Front. Plant Sci.* **8**, 1195 (2017).

15. J. H. Jun, E. Fiume, J. C. Fletcher, The CLE family of plant polypeptide signaling molecules. *Cell. Mol. Life Sci.* **65**, 743–755 (2008).

16. J. Kang, X. Wang, T. Ishida, E. Grienenberger, Q. Zheng, J. Wang, Y. Zhang, W. Chen, M. Chen, X.-F. Song, C. Wu, Z. Hu, L. Jia, C. Li, C.-M. Liu, J. C. Fletcher, S. Sawa, G. Wang, A group of CLE peptides regulates de *novo* shoot regeneration in *Arabidopsis thaliana*. *New Phytol.* **235**, 2300–2312 (2022).

17. S. Nakagami, T. Aoyama, Y. Sato, T. Kajiwara, T. Ishida, S. Sawa, CLE3 and its homologues share overlapping functions in the modulation of lateral root formation through CLV1 and BAM1 in *Arabidopsis thaliana*. *Plant J.* **6**, 1176–1191 (2023).

18. N. Czyzewicz, C.-L. Shi, L. D. Vu, B. Van De Cotte, C. Hodgman, M. A. Butenko, I. D. Smet, Modulation of *Arabidopsis* and monocot root architecture by CLAVATA3/EMBRYO SURROUNDING REGION 26 peptide. *J. Exp. Bot.* **66**, 5229–5243 (2015).

19. J. H. Jun, E. Fiume, A. H. K. Roeder, L. Meng, V. K. Sharma, K. S. Osmont, C. Baker, C. M. Ha, E. M. Meyerowitz, L. J. Feldman, J. C. Fletcher, Comprehensive analysis of *CLE* polypeptide signaling gene expression and overexpression activity in *Arabidopsis*. *Plant Physiol.* **154**, 1721–1736 (2010).

20. J. Zuo, Q. W. Niu, N. H. Chua, An estrogen receptor-based transactivator XVE mediates highly inducible gene expression in transgenic plants. *Plant J.* **24**, 265–273 (2000).

21. A. Kereszt, P. Mergaert, J. Montiel, G. Endre, É. Kondorosi, Impact of plant peptides on symbiotic nodule development and functioning. *Front. Plant Sci.* **9**, 1026 (2018).

22. K. Ohyama, H. Shinohara, M. Ogawa-Ohnishi, Y. Matsubayashi, A glycopeptide regulating stem cell fate in *Arabidopsis thaliana*. *Nat. Chem. Biol.* **5**, 578–580 (2009).

23. T. Araya, M. Miyamoto, J. Wibowo, A. Suzuki, S. Kojima, Y. N. Tsuchiya, S. Sawa, H. Fukuda, N. von Wirén, H. Takahashi, CLE-CLAVATA1 peptide-receptor signaling module regulates the expansion of plant root systems in a nitrogen-dependent manner. *Proc. Natl. Acad. Sci. U.S.A.* **111**, 2029–2034 (2014).

24. V. K. Sharma, J. C. Fletcher, Maintenance of shoot and floral meristem cell proliferation and fate. *Plant Physiol.* **129**, 31–39 (2002).

25. Y. Stahl, R. H. Wink, G. C. Ingram, R. Simon, A signaling module controlling the stem cell niche in *Arabidopsis* root meristems. *Curr. Biol.* **19**, 909–914 (2009).

26. Y. Stahl, S. Grabowski, A. Bleckmann, R. Kühnemuth, S. Weidtkamp-Peters, K. G. Pinto, G. K. Kirschner, J. B. Schmid, R. H. Wink, A. Hülsewede, S. Felekyan, C. A. M. Seidel, R. Simon, Moderation of *Arabidopsis* Root Stemness by CLAVATA1 and ARABIDOPSIS CRINKLY4 receptor kinase complexes. *Curr. Biol.* **23**, 362–371 (2013).

27. S. Okamoto, T. Suzuki, M. Kawaguchi, T. Higashiyama, Y. Matsubayashi, A comprehensive strategy for identifying long-distance mobile peptides in xylem sap. *Plant J.* **84**, 611–620 (2015).

28. S. Okamoto, A. Kawasaki, Y. Makino, T. Ishida, S. Sawa, Long-distance translocation of CLAVATA3/ESR-related 2 peptide and its positive effect on roots sucrose status. *Plant Physiol.* **189**, 2357–2367 (2022).

29. R. Tabata, K. Sumida, T. Yoshii, K. Ohyama, H. Shinohara, Y. Matsubayashi, Perception of root-derived peptides by shoot LRR-RKs mediates systemic N-demand signaling. *Science* **346**, 343–346 (2014).

30. C. W. Lim, Y. W. Lee, C. H. Hwang, Soybean nodule-enhanced CLE peptides in roots act as signals in GmNARK-mediated nodulation suppression. *Plant Cell Physiol.* **52**, 1613–1627 (2011).

31. V. Mortier, G. Den Herder, R. Whitford, W. Van de Velde, S. Rombauts, K. D'haeseleer, M. Holsters, S. Goormachtig, CLE peptides control *Medicago truncatula* nodulation locally and systemically. *Plant Physiol.* **153**, 222–237 (2010).

32. D. E. Reid, B. J. Ferguson, P. M. Gresshoff, Inoculation- and nitrate-induced CLE peptides of soybean control NARK-dependent nodule formation. *Mol. Plant Microbe Interact.* **24**, 606–618 (2011).

33. D. Ma, S. Endo, S. Betsuyaku, A. Shimotohno, H. Fukuda, CLE2 regulates light-dependent carbohydrate metabolism in *Arabidopsis* shoots. *Plant Mol. Biol.* **104**, 561–574 (2020).

34. M. G. Mitchum, X. Liu, Peptide effectors in phytonematode parasitism and beyond. *Annu. Rev. Phytopathol.* **60**, 97–119 (2022).

35. J. Wang, A. Dhroso, X. Liu, T. J. Baum, R. S. Hussey, E. L. Davis, X. Wang, D. Korkin, M. G. Mitchum, Phytonematode peptide effectors exploit a host post-translational trafficking mechanism to the ER using a novel translocation signal. *New Phytol.* **229**, 563–574 (2021).

36. G. Huang, R. Dong, R. Allen, E. L. Davis, T. J. Baum, R. S. Hussey, A root-knot nematode secretory peptide functions as a ligand for a plant transcription factor. *Mol. Plant Microbe Interact.* **19**, 463–470 (2006).

37. W. B. Rutter, T. Hewezi, T. R. Maier, M. G. Mitchum, E. L. Davis, R. S. Hussey, T. J. Baum, Members of the *Meloidogyne* Avirulence protein family contain multiple plant ligand-like motifs. *Phytopathology* **104**, 879–885 (2014).

38. G. Huang, R. Allen, E. L. Davis, T. J. Baum, R. S. Hussey, Engineering broad root-knot resistance in transgenic plants by RNAi silencing of a conserved and essential root-knot nematode parasitism gene. *Proc. Nati. Acad. Sci. U.S.A.* **103**, 14302–14306 (2006).

39. B. Absmanner, R. Stadler, U. Z. Hammes, Phloem development in nematode-induced feeding sites: The implications of auxin and cytokinin. *Front. Plant Sci.* **4**, 241 (2013).

40. M. Barcala, A. García, J. Cabrera, S. Casson, K. Lindsey, B. Favery, G. García-Casado, R. Solano, C. Fenoll, C. Escobar, Early transcriptomic events in microdissected *Arabidopsis* nematode-induced giant cells. *Plant J.* **61**, 698–712 (2010).

41. C. D. Dowd, D. Chronis, Z. S. Radakovic, S. Siddique, T. Schmülling, T. Werner, T. Kakimoto, F. M. W. Grundler, M. G. Mitchum, Divergent expression of cytokinin biosynthesis, signaling and catabolism genes underlying differences in feeding sites induced by cyst and root-knot nematodes. *Plant J.* **92**, 211–228 (2017).

42. A. Islam, C. F. Mercer, S. Leung, P. P. Dijkwel, M. T. McManus, Transcription of biotic stress associated genes in white clover (*Trifolium repens L.*) Differs in response to cyst and root-knot nematode infection. *PLOS ONE* **10**, e0137981 (2015).

43. S. J. Shah, M. S. Anjam, B. Mendy, M. A. Anwer, S. S. Habash, J. L. Lozano-Torres, F. M. W. Grundler, S. Siddique, Damage-associated responses of the host contribute to defence against cyst nematodes but not root-knot nematodes. *J. Exp. Bot.* **68**, 5949–5960 (2017).

44. M. Hayashi-Tsugane, M. Kawaguchi, *Lotus japonicus* HAR1 regulates root morphology locally and systemically under a moderate nitrate condition in the absence of rhizobia. *Planta* **255**, 95 (2022).

45. R. Nishimura, M. Hayashi, G. J. Wu, H. Kouchi, H. Imaizumi-Anraku, Y. Murakami, S. Kawasaki, S. Akao, M. Ohmori, M. Nagasawa, K. Harada, M. Kawaguchi, HAR1 mediates systemic regulation of symbiotic organ development. *Nature* **420**, 426–429 (2002).

46. J. Wopereis, E. Pajuelo, F. B. Dazzo, Q. Jiang, P. M. Gresshoff, F. J. de Bruijn, J. Stougaard, K. Szczyglowski, Short root mutant of *Lotus japonicus* with a dramatically altered symbiotic phenotype. *Plant J.* **23**, 97–114 (2000).

47. E. Huault, C. Laffont, J. Wen, K. S. Mysore, P. Ratet, G. Duc, F. Frugier, Local and systemic regulation of plant root system architecture and symbiotic nodulation by a receptor-like kinase. *PLOS Genet.* **10**, e1004891 (2014).

48. K. Chapman, M. Taleski, H. A. Ogilvie, N. Imin, M. A. Djordjevic, *CEP–CEPR1* signalling inhibits the sucrose-dependent enhancement of lateral root growth. *J. Exp. Bot.* **70**, 3955–3967 (2019).

49. I. Dimitrov, F. E. Tax, Lateral root growth in *Arabidopsis* is controlled by short and long distance signaling through the LRR RLKs XIP1/CEPR1 and CEPR2. *Plant Signal. Behav.* **13**, e1489667 (2018).

50. H. Miyazawa, E. Oka-Kira, N. Sato, H. Takahashi, G. J. Wu, S. Sato, M. Hayashi, S. Betsuyaku, M. Nakazono, S. Tabata, K. Harada, S. Sawa, H. Fukuda, M. Kawaguchi, The receptor-like kinase KLAVIER mediates systemic regulation of nodulation and non-symbiotic shoot development in *Lotus japonicus*. *Development* **137**, 4317–4325 (2010).

51. Z. Luo, J. Lin, Y. Zhu, M. Fu, X. Li, F. Xie, NLP1 reciprocally regulates nitrate inhibition of nodulation through SUNN-CRA2 signaling in *Medicago truncatula*. *Plant Commun.* **2**, 100183 (2021).

52. H. Nishida, S. Tanaka, Y. Handa, M. Ito, Y. Sakamoto, S. Matsunaga, S. Betsuyaku, K. Miura, T. Soyano, M. Kawaguchi, T. Suzaki, A NIN-LIKE PROTEIN mediates nitrate-induced control of root nodule symbiosis in *Lotus japonicus*. *Nat. Commun.* **9**, 499 (2018).

53. H. Nishida, S. Nosaki, T. Suzuki, M. Ito, T. Miyakawa, M. Nomoto, Y. Tada, K. Miura, M. Tanokura, M. Kawaguchi, T. Suzaki, Different DNA-binding specificities of NLP and NIN transcription factors underlie nitrate-induced control of root nodulation. *Plant Cell* **33**, 2340–2359 (2021).

54. W. Dong, Y. Wang, H. Takahashi, CLE-clavata1 signaling pathway modulates lateral root development under sulfur deficiency. *Plan. Theory* **8**, 103 (2019).

55. D. Zhao, Y. You, H. Han, X. Zhu, Y. Wang, Y. Duan, Y. Xuan, L. Chen, The role of sugar transporter genes during early infection by root-knot nematodes. *Int. J. Mol. Sci.* **19**, 302 (2018).

56. A. Martínez-Medina, C. M. Mbaluto, A. Maedicke, A. Weinhold, F. Vergara, N. M. van Dam, Leaf herbivory counteracts nematode-triggered repression of jasmonate-related defenses in tomato roots. *Plant Physiol.* **187**, 1762–1778 (2021).

57. B. J. De Young, K. L. Bickle, K. J. Schrage, P. Muskett, K. Patel, S. E. Clark, The CLAVATA1-related BAM1, BAM2 and BAM3 receptor kinase-like proteins are required for meristem function in *Arabidopsis*. *Plant J.* **45**, 1–16 (2006).

58. K. Fisher, S. Turner, PXY, a receptor-like kinase essential for maintaining polarity during plant vascular-tissue development. *Curr. Biol.* **17**, 1061–1066 (2007).

59. A. Kinoshita, S. Betsuyaku, Y. Osakabe, S. Mizuno, S. Nagawa, Y. Stahl, R. Simon, K. Yamaguchi-Shinozaki, H. Fukuda, S. Sawa, RPK2 is an essential receptor-like kinase that transmits the CLV3 signal in *Arabidopsis*. *Development* **137**, 4327 (2010).

60. Y. L. Yamaguchi, T. Ishida, M. Yoshimura, Y. Imamura, C. Shimaoka, S. Sawa, A collection of mutants for CLE-peptide-encoding genes in *Arabidopsis* generated by CRISPR/Cas9-mediated gene targeting. *Plant Cell Physiol.* **58**, 1848–1856 (2017).

61. A. Imoto, M. Yamada, T. Sakamoto, A. Okuyama, T. Ishida, S. Sawa, M. Aida, A ClearSee-based clearing protocol for 3D visualization of *Arabidopsis thaliana* embryos. *Plan. Theory* **10**, 190 (2021).

62. J. D. Pédelacq, S. Cabantous, T. Tran, T. C. Terwilliger, G. S. Waldo, Engineering and characterization of a superfolder green fluorescent protein. *Nat. Biotechnol.* **24**, 79–88 (2006).

63. D. S. Favero, A. Kawamura, M. Shibata, A. Takebayashi, J.-H. Jung, T. Suzuki, K. E. Jaeger, T. Ishida, A. Iwase, P. A. Wigge, M. M. Neff, K. Sugimoto, AT-hook transcription factors restrict petiole growth by antagonizing PIFs. *Curr. Biol.* **30**, 1454–1466.e6 (2020).

64. H. Nishiyama, B. T. Ngan, S. Nakagami, C. Ejima, T. Ishida, S. Sawa, Protocol for root-knot nematode culture by a hydroponic system and nematode inoculation to *Arabidopsi*. *Japanese J. Nematol.* **45**, 45–49 (2015).

65. C. G. N. Turnbull, J. P. Booker, H. M. O. Leyser, Micrografting techniques for testing long-distance signalling in *Arabidopsis*. *Plant J.* **32**, 255–262 (2002).

66. D. Gaidatzis, A. Lerch, F. Hahne, M. B. Stadler, QuasR: Quantification and annotation of short reads in R. *Bioinformatics* **31**, 1130–1132 (2015).

67. J. Sun, T. Nishiyama, K. Shimizu, K. Kadota, TCC: An R package for comparing tag count data with robust normalization strategies. *BMC Bioinformatics* **14**, 219 (2013).

68. T. Kondo, T. Nakamura, K. Yokomine, Y. Sakagami, Dual assay for MCLV3 activity reveals structure—Activity relationship of CLE peptides. *Biochem. Biophys. Res. Commun.* **377**, 312–316 (2008).

69. H. Shinohara, M. Ogawa, Y. Sakagami, Y. Matsubayashi, Identification of ligand binding site of phytosulfokine receptor by on-column photoaffinity labeling. *J. Biol. Chem.* **282**, 124–131 (2007).

70. R. Wang, K. Guegler, S. T. LaBrie, N. M. Crawford, Genomic analysis of a nutrient response in *Arabidopsis* reveals diverse expression patterns and novel metabolic and potential regulatory genes induced by nitrate. *Plant Cell* **12**, 1491–1509 (2000).
